# Bidirectional Energy Flow in the Photosystem II Supercomplex

**DOI:** 10.1021/acs.jpcb.4c02508

**Published:** 2024-08-14

**Authors:** Cristina Leonardo, Shiun-Jr Yang, Kaydren Orcutt, Masakazu Iwai, Eric A. Arsenault, Graham R. Fleming

**Affiliations:** † Department of Chemistry, University of California, Berkeley, California 94720, United States; ‡ Molecular Biophysics and Integrated Bioimaging Division, Lawrence Berkeley National Laboratory, Berkeley, California 94720, United States; § Department of Chemistry, University of California, Berkeley, Berekeley, California 94720, United States; ∥ Kavli Energy Nanoscience Institute at Berkeley, Berkeley, California 94720, United States; ⊥ Department of Plant and Microbial Biology, University of California, Berkeley, Berekeley, California 94720, United States

## Abstract

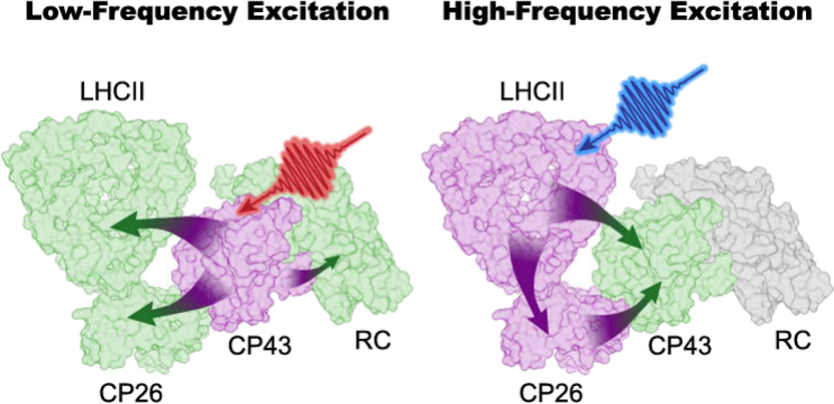

The water-splitting capability of Photosystem II (PSII) of plants and green algae requires the system to balance efficient light harvesting along with effective photoprotection against excitation in excess of the photosynthetic capacity, particularly under the naturally fluctuating sunlight intensity. The comparatively flat energy landscape of the multicomponent structure, inferred from the spectra of the individual pigment–protein complexes and the rather narrow and featureless absorption spectrum, is well known. However, how the combination of the required functions emerges from the interactions among the multiple components of the PSII supercomplex (PSII-SC) cannot be inferred from the individual pigment–protein complexes. In this work, we investigate the energy transfer dynamics of the C_2_S_2_-type PSII-SC with a combined spectroscopic and modeling approach. Specifically, two-dimensional electronic-vibrational (2DEV) spectroscopy provides enhanced spectral resolution and the ability to map energy evolution in real space, while the quantum dynamical simulation allows complete kinetic modeling of the 210 chromophores. We demonstrate that additional pathways emerge within the supercomplex. In particular, we show that excitation energy can leave the vicinity of the charge separation components, the reaction center (RC), faster than it can transfer to it. This enables activatable quenching centers in the periphery of the PSII-SC to be effective in removing excessive energy in cases of overexcitation. Overall, we provide a quantitative description of how the seemingly contradictory functions of PSII-SC arise from the combination of its individual components. This provides a fundamental understanding that will allow further improvement of artificial solar energy devices and bioengineering processes for increasing crop yield.

## 1 Introduction

Solar energy is arguably the most valuable energy source on Earth, yet it is ineffectively exploited by humans. Control and regulation of solar energy conversion processes remain major challenges. One promising approach toward the improvement of solar utilization is through the development and application of bioinspired design, as natural light-harvesting is the paradigm for solar energy conversion processes with high quantum efficiency. In the early stages of photosynthesis, natural photosynthetic organisms take advantage of large antenna systems to harvest solar energy at a rapid average rate to efficiently convert the energy to produce electrons, which drive the subsequent chemical reactions. Most systems have evolved an energy “funnel” as the most effective way to boost efficiency.^1^ However, it has been suggested that no energy funnel to the reaction center (RC) is present in photosystem II (PSII) of a large antenna system,^3−7^ which can still surprisingly achieve near unit quantum efficiency at low light levels. On the other hand, the high oxidative power of the PSII-RC, which is required for water splitting, can easily lead to reactive oxygen species formation when there is excessive sunlight.^2^ Thus, the high quantum efficiency at low light levels must be balanced with an effective photoprotective system at high light levels, especially in natural environments where sunlight intensity fluctuates throughout the diurnal cycle. The importance of a responsive photoprotective system in determining crop yield has recently been demonstrated.^10^ In addition to the multiple complex components that are required to switch between efficient charge separation and photoprotective modes, the energy transfer network in light-harvesting systems also must be designed to work with these components. Therefore, even though the energy transfer network does not change quickly under different light intensities, a deeper understanding of the energy flow in PSII could indicate pathways to further improvement. In particular, understanding the working mechanism underlying the evolutionarily chosen flat energy landscape for effective light harvesting and photoprotection is crucial.

**Figure 1:**
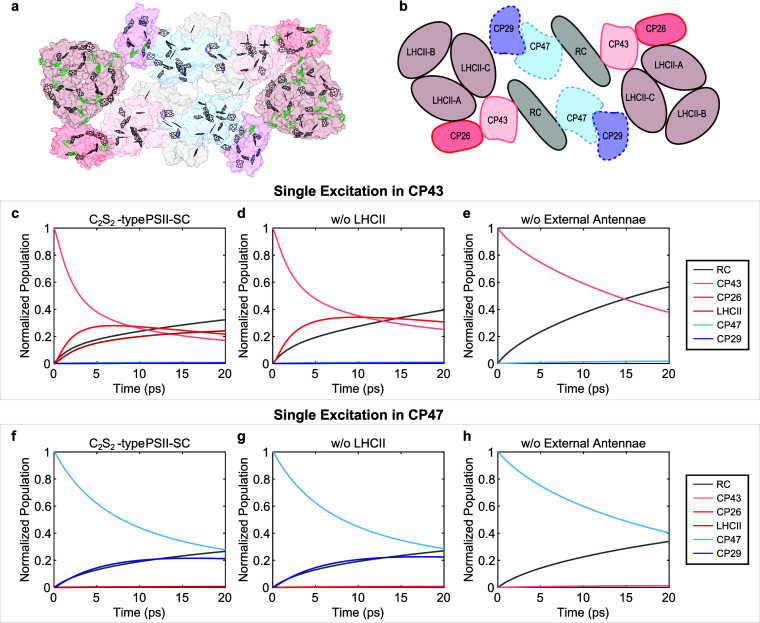
(a) Pigment arrangement in the C_2_S_2_-type PSII-SC from spinach (PDB: 3JCU)^13^: Chls *a* are in black; Chls *b* are in green; and pheophytins are in magenta. (b) Schematic of the PSII-SC protein subunits. Proteins on the D1 side are represented by solid lines, and those on the D2 side are represented by dashed lines. Simulated excitation population dynamics of (c) the complete C_2_S_2_-type PSII-SC (d) PSII-SC without LHCII and (e) without LHCII, CP26 and CP29 upon single excitation in CP43. Simulated excitation population dynamics of (f) the complete C_2_S_2_-type PSII-SC (g) PSII-SC without LHCII and (h) without LHCII, CP26 and CP29 upon single excitation in CP47.

In the thylakoid membrane, PSII is bound with light-harvesting complex II (LHCII) trimers to form PSII-LHCII supercomplexes in a ratio that depends on the acclimated condition. The C_2_S_2_-type PSII-LHCII supercomplex (referred to as the PSII-SC in the following) is the dominant form in high-light conditions,^11,12^ where photoprotection is crucial. The arrangement of the chromophores and protein subunits in the C_2_S_2_-type PSII-SC from spinach is shown in Figure 1a,b. The high-resolution cryo-EM structure^13^ shows that PSII-SC is a dimeric complex with 4 pheophytins and 206 chlorophylls (Chls), of which 156 are Chls *a* and 50 are Chls *b*. Each monomer contains one RC, consisting of two branches (D1 and D2), two core antennae (CP43 and CP47), two minor antennae (CP26 and CP29), and one strongly bound LHCII. The subunit containing the RC, CP43, and CP47 is referred to as the PSII core complex (PSII-CC). Among the antennae, LHCII, CP26, and CP43 are in proximity to the D1 branch, the branch that actively performs charge separation,^14,15^ while CP29 and CP47 are on the D2 side of the complex.

The study of electronic energy transfer (EET) dynamics in the complete PSII-SC is challenging. These dynamics typically occur within tens of femtoseconds to a few hundred picoseconds, requiring the use of ultrafast spectroscopic techniques. However, significant spectral congestion due to the large number of Chls present in the PSII-SC challenges the currently available technology. As a result, most studies focus on the isolated complexes and smaller subunits of the PSII-SC where the numbers of convoluted processes are reduced.^16−23^ These studies provide insight into EET pathways existing within the subunits but are insufficient for obtaining a complete description of energy flow within the PSII-SC relevant to its functions. In the past, fluorescence lifetime studies have been reported for the entire supercomplex.^24−28^ However, the energy flow between different subunits cannot be directly inferred from these experiments. Moreover, the uncertainty in kinetic modeling based on the fitting of the fluorescence decays cannot be avoided. In addition to the experimental studies, many theoretical simulations have been performed to understand the dynamics within different isolated PSII subunits.^5,7,29−31^ While these works provide a detailed understanding of the EET dynamics in each subunit, a simulation of the whole PSII-SC is required to connect the microscopic interactions to its ability to balance efficiency and photoprotection. Based on these works, Bennett et al. constructed the structure-based model of the PSII-SC mentioned earlier.^32^ However, the structural information available at the time was not at high resolution (∼12 Å) and can be used only to determine relative orientations and approximate distances between individual proteins. Valkunas and co-workers proposed a model that takes into account the heterogeneity of the PSII-SC by introducing excitation diffusion parameters.^33^ The parameters, extracted from fitting fluorescence decays, reveal the connectivity between different protein subunits but do not provide information about specific EET pathways.

To investigate the EET dynamics in the PSII-SC, we rely on a combination of two-dimensional electronic-vibrational (2DEV) spectroscopy^8,9^ and a structure-based dynamical simulation to characterize the early time (<20 ps) interprotein EET dynamics within the C_2_S_2_-type PSII-SC from spinach. In 2DEV spectroscopy, the simultaneous resolution along the visible excitation and the mid-infrared (IR) probe axes reduces the spectral congestion that limits the resolution of other ultrafast spectroscopic techniques, thus enabling the study of complex systems such as the PSII-SC.^34−40^ Specifically, the mid-IR detection provides a means to distinguish different protein subunits, as the localized vibrational modes are sensitive to the protein surrounding. Meanwhile, the high-resolution cryo-EM structure^13^ allows a more accurate description of the interactions between the pigments, which leads to a more accurate kinetic model. Together, they reveal the interprotein EET pathways only present in the entire PSII-SC that are crucial for efficient energy conversion and effective photoprotection.

## 2 Methods

### 2.1 Sample Preparation

All procedures for sample preparation were performed in the dark to minimize exposure to light as much as possible. We prepared PSII-enriched membranes from spinach leaves according to the previous literature^41,42^ with some modifications as described previously.^38,39^ For preparing the C_2_S_2_-type PSII-SC, the PSII-enriched membranes (0.5 mg of Chl/mL) were solubilized with 1.0% (w/v) α-DDM (n-dodecyl-α-D-maltopyranoside, Anatrace) in a buffer containing 25 mM MES-NaOH (pH 6.0) for 30 min on ice. The solubilized membranes were then centrifuged at 21,000 × *g* for 5 min at 4 °C. The supernatants were loaded onto sucrose gradients (each concentration overlaid with the denser one: 2.1 mL of 0.1, 0.4, 0.7, 1.0, and 1.3 M sucrose and 0.5 mL of 1.8 M sucrose at the bottom) in ultracentrifuge tubes (14 × 89 mm, Beckman Coulter). The sucrose gradients contained 0.03% (w/v) α-DDM buffer as described above. Centrifugation was performed at 154,300 × *g* for 24 h at 4 °C (SW 41 Ti swinging-bucket rotor, Beckman Coulter). The separated bands were collected dropwise from the bottom of the tube. The collected fraction containing the C_2_S_2_-type PSII-SC was concentrated using a centrifugal filter (100 K MWCO). The concentrated sample was diluted with a buffer containing 25 mM MES-NaOH (pH 6.0), 10 mM NaCl, 3 mM CaCl_2_, 400 mM sucrose, and 0.03% α-DDM prepared with D_2_O. The concentrated C_2_S_2_-type PSII-SC was flash-frozen and stored at −80 °C until the 2DEV measurements. The preparation of isolated LHCII follows the procedure in the work of Arsenault et al.^36^ with the only exception being the final resuspension step, which is done with the same buffer used in the preparation of the C_2_S_2_-type PSII-SC.

### 2.2 2DEV Measurements

The details of the 2DEV setup can be found elsewhere.^37−39^ The reported PSII-SC 2DEV data were collected in two separate measurements at 77 K (Figure S1). For the first measurement, the excitation pulses were centered at 665 nm with a fwhm of 70 nm, and the sample was diluted with glycerol to have an optical density of ∼0.6 at 675 nm. For the second measurement, the excitation pulses were centered at 630 nm with a full width at half-maximum (fwhm) of 55 nm, and the sample was diluted to have an optical density of ∼1.0 at 650 nm. For isolated LHCII, the 2DEV measurement was performed at 77 K with excitation pulses centered at 655 nm with a fwhm of 55 nm. The sample was diluted with glycerol to have an optical density of ∼0.8 at 675 nm. For all measurements, the optical path length was ∼200 μm and the excitation pulses were compressed to 15–20 fs. Both pulses were focused to a spot size of ∼200 μm with a combined energy of ∼80 nJ. The detection pulse was centered at 5900 nm and spanned over 5500–6300 nm. The instrument response is estimated to be ∼90 fs from cross-correlation between the visible and mid-IR pulses. The time delays between the second visible pump and the IR probe are the same for all measurements: from 0 to 1.04 ps with steps of 20 fs; from 1.14 to 20 ps with steps of 100 fs; and from 25 to 100 ps with steps of 5 ps. The repetition rate of the source laser is set to 1 kHz.

### 2.3 Lifetime Density Analysis (LDA)

LDA approximates the data evolution as a sum of hundreds of exponentials to retrieve the corresponding amplitudes (*x*_*j*_(τ_*j*_, λ)), producing lifetime density maps (LDM)^43^:
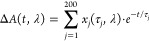
1

Despite LDA resulting in high uncertainty in the time constants, the ability to handle hundreds of exponential trends with little initial assumptions is an important advantage over the widely used global analysis.^44^ The data analysis was performed using pyLDM.^45^ Regularization of the minimization process is always applied to obtain reliable LDMs.^45^ A low regularization hyperparameter α, which corresponds to higher LDM amplitudes and narrower lifetime distributions, is initially adopted. However, low values of α have a higher chance to return artifacts. This is true independently of the noise level, as is observed also for the noise-free simulated population evolution. Figure S2a shows an example of LDM obtained for a low α, showing that the artifact appears as a satellite peak with opposite amplitude compared to the main lifetimes. To identify the artifacts, exponential fits of the LDM obtained for the regularization parameter α = 0.1 are performed (see Figure S2 and Table S3). Based on the fitting, we select the optimal hyperparameter α that returns artifact-free LDMs. In particular, we find that α = 3 provides the best results for the LDA of all experimental data and simulated population evolution (Figure S2b). Only for the simulated LHCII excitation population, an α = 1 was adopted. We note that a higher hyperparameter α leads to wider lifetime distributions (Figure S2a–c). The broadening of the lifetime distributions, therefore, is merely a consequence of the analysis.^45^

### 2.4 EET Dynamics Simulation

The simulations were performed based on a modified version of the structure-based model proposed in the work of Bennett et al., where detailed simulation procedures can be found.^32^ The differences between the model used in this work and the work of Bennett et al. are listed and discussed here. The parameters used for the simulations can be found in Tables S1 and S2.

1. The interprotein pigment couplings were calculated based on the cryo-EM structure of the C_2_S_2_-type PSII supercomplex extracted from spinach (PDB: 3JCU),^13^ and the TrEsp method was used instead of applying the point dipole approximation.^46^ The atomic transition charges of Chls *a*, Chls *b* and pheophytins *a* were obtained from the literature^46−48^ and scaled to match the transition dipole moments listed in Table S1.
2. The CP29 Hamiltonian, originally represented by the LHCII monomer Hamiltonian in Bennett’s model, was described by a new Hamiltonian proposed by Mascoli et al.^31^ The new CP29 Hamiltonian was constructed based on isolated CP29, in which C616 is absent due to purification. Therefore, the C616 is not included in the CP29 Hamiltonian. Additionally, the 13-state Hamiltonian contains C614, which is absent in the 3JCU structure and is, therefore, deleted from the Hamiltonian. Currently, there is no semiempirical Hamiltonian for CP26. Due to the spectral similarity between CP26 and CP29,^49^ the CP26 Hamiltonian is represented by the CP29 Hamiltonian with C614 included, which is present in CP26 in the 3JCU structure.
3. The line-broadening functions were calculated for 77 K to match the experimental conditions. Unlike the calculation for 300 K (both in this work and Bennett’s model), the line shape functions for the core components (RC, CP43, and CP47) do not converge in the time domain without a dephasing term, which was originally included in the work of Renger and co-workers^29,50^ but omitted in Bennett’s model. At 300 K, the effect of the dephasing term is negligible due to stronger electron–phonon interaction, and can therefore be omitted. For the calculations at 77 K, the term is required to ensure the convergence of line-shape functions. In our simulations, the dephasing term was included for the core components, and the dephasing time was taken as 1 ps. Different values were tested and the effect is negligible compared to inhomogeneous broadening.

The population of each state at each time point was then calculated based on the hybrid rate matrix (combining generalized-Förster and modified-Redfield rates, see ref (32)) with the following equation:
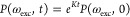
2where *P*(ω_exc_, *t*) is the excitation-dependent population, *K* is the hybrid rate matrix and *P*(ω_exc_, 0) is the excitation frequency-dependent initial population calculated based on the integrated absorption of individual excitonic states within a 10 cm^–1^ range (centered at each defined excitation frequency). To be more specific, the population in an excitonic state is linearly proportional to its absorption in the integrated frequency range and later normalized according to the absorption strength of all excitonic states. The population evolution of each protein was calculated by converting the exciton population to the Chl population and summing over all of the Chls within each protein. Simulation of single excitation (Figure 1c–h) was performed by assigning excitation to one single Chl as the initial population, C509 for CP43 and C611 for CP47, both of which are at the center of each protein. We note that, in principle, the charge separation dynamics can be included in the rate matrix by incorporating the time scales obtained from the fitting of experimental data. However, such fitting has been demonstrated to be problematic as different models can provide equally good fits.^32^ Therefore, instead of including empirical charge separation lifetimes in our model, it is assumed that charge separation occurs infinitely faster than EET out of RC components. Such an approximation is not only consistent with the transfer-to-trap limited model^19,28,29^ reported in the literature but it has also been applied to another model^33^ which was able to reproduce experimental results with excellent agreement. While we do not expect the approximation to change the overall dynamics of energy transfer, especially at early waiting times, future improvements can be made by properly incorporating descriptions of charge transfer dynamics into the model.

We note that Bennett et al. also proposed the “domain model”, in which it is assumed that intradomain EET is fast enough to allow thermal equilibrium within each domain before interdomain EET occurs. They showed that the dynamics predicted by the hybrid model and the domain model share great similarities. However, in our calculations, the dynamics differ dramatically when thermal equilibrium is assumed under cryogenic conditions (77 K). In contrast, the room temperature (300 K) simulations, which most likely allow faster equilibration, generate similar results with both models, as described in the work of Bennett et al. For consistency, the population evolution of both conditions was calculated based on the hybrid rate matrix instead of the domain-to-domain transfer rate matrix.

The simulation of quenching probability (Figure S3) with activated quenchers was performed by connecting additional sinks (where reverse transfer is prohibited) to the Chls that are suspected of being responsible for EET to carotenoids, C602–C603 and C610–C612.^51,52^ The rate for EET from these Chls to carotenoid is set to be (200 fs)^−1^, which is similar to the values reported in the literature.^53,54^ The quenching probability is then defined as the ratio between the population in the sinks and the RC at the long time limit. In each simulation, two quenchers were each placed in the same protein subunit of the two PSII-SC monomers. All simulations were performed for both 77 and 300 K.

## 3 Results and Discussion

### 3.1 Kinetic Network Within the Complete PSII-SC

The subunits of PSII-SC cooperatively form an EET network that initiates photosynthesis. In particular, the PSII-SC functions strongly rely on the interprotein EET pathways that originate from its multicomponent structure. To understand how the multiunit construction relates to the photosynthetic functions of PSII, it is necessary to study the complete PSII-SC as some of the crucial pathways are only present when all the subunits are connected. Furthermore, the presence of these pathways alters the significance of the pathways in the isolated subunits. We demonstrate this by comparing the EET dynamics obtained from structure-based simulations for isolated PSII-SC subunits of different sizes. Briefly, we adapt the kinetic model of the PSII-SC proposed by Bennett et al.^32^ and follow their methods to reconstruct a new structure-based model for the C_2_S_2_-type PSII-SC based on the state-of-the-art high-resolution structure (protein data bank: 3JCU).^13^ The kinetic model was used to produce excitation population evolution, which was coarse-grained to allow focus on the interprotein EET pathways. Figure 1c–h shows the evolution of the excitation population in the PSII-SC subunits with different sizes of the antenna system upon the excitation of the core, CP43 and CP47. While the initial excitation is placed in the same Chl in either CP43 or CP47, the absence of LHCII or the minor antennae alters the EET dynamics. For example, the absence of LHCII results in faster RC growth and slower CP43 decay, while the absence of all external antennae results in even more obvious shifts in time scales of the EET dynamics. The different traces indicate that the time scales observed in the isolated smaller subunits of the PSII-SC do not necessarily reflect the EET pathways relevant in the native environment, where PSII exists in the form of the PSII-SC. This is because the presence of additional subunits opens up new pathways for energy flow so that the time scales observed in complexes lacking the full complement of components of the PSII-SC do not necessarily stay the same in the more complex EET network in the complete PSII-SC. Additionally, potential structural changes caused by the removal of protein subunits, not taken into account in the simulations, can further alter the EET pathways present in the complete system. These stress the importance of studying the entire PSII-SC as the investigation of smaller subunits can lead to inaccurate descriptions of the relevant EET pathways that initiate photosynthesis in nature.

### 3.2 Bidirectional Energy Transfer

**Figure 2:**
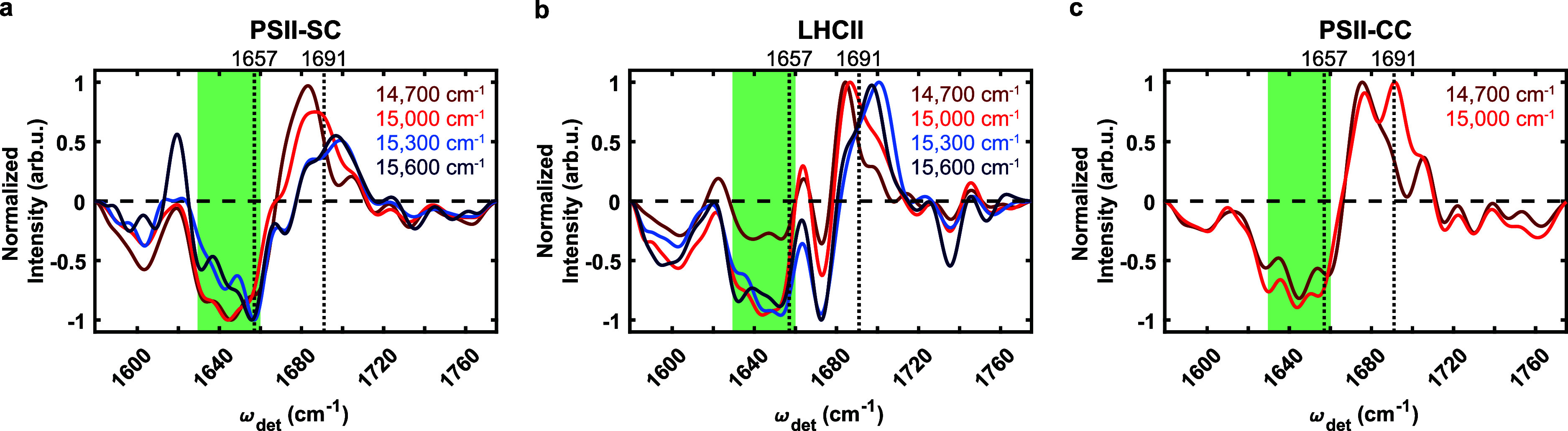
2DEV slices at 14,700 cm^–1^ (dark red), 15,000 cm^–1^ (red), 15,300 cm^–1^ (blue), and 15,600 cm^–1^ (dark blue) for (a) the PSII-SC, (b) LHCII, and (c) the PSII-CC. The vertical dotted lines correspond to 1657 and 1691 cm^–1^. The green areas cover the region of 1630–1660 cm^–1^. The 2DEV slices of the PSII-CC were reproduced with permission from ref (39). Available under a CC BY license. Copyright (2022) Authors.

**Figure 3:**
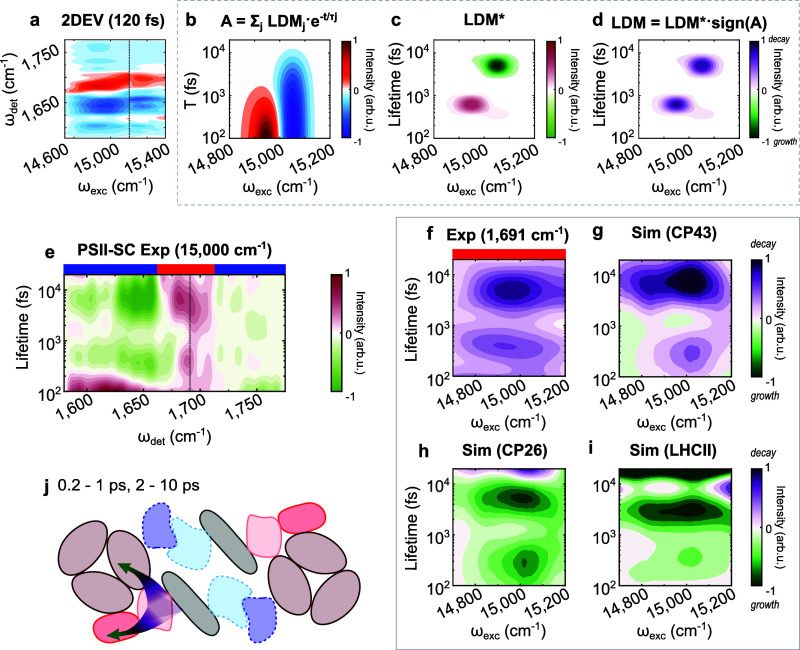
(a) 2DEV spectrum of the PSII-SC at time delays of *T* = 120 fs. Positive (red) and negative (blue) amplitudes are ground state bleach (GSB) and excited state absorption (ESA) signals, respectively. The dashed line marks the separation of two experiments (see Experimental). (b) Simulated evolution of the excitation population, where both the GSB and ESA features decay. (c) Corresponding LDM showing different signs due to the sign difference between GSB and ESA. (d) LDM multiplied by signal sign, where positive amplitudes indicate decays. (e) LDM of the 2DEV data averaged over 14,800–15,200 cm^–1^: vertical dashed line at 1691 cm^–1^; blue (red) bars on top refer to ESA (GSB) signatures. Excitation-dependent LDM of (f) the GSB at 1691 cm^–1^ from the 2DEV data and the simulated population evolution of (g) CP43, (h) CP26, and (i) LHCII. Positive amplitudes (purple) indicate decay; negative amplitudes (green) indicate growth. (j) Schematic of the corresponding interprotein EET at 0.2–1 and 2–10 ps.

In order to understand the complex EET network in PSII-SC, we combine 2DEV spectroscopy and simulation. Figure 2 shows the 2DEV spectral slices along the detection axis of PSII-SC, LHCII, and PSII-CC at specific excitation frequencies at an early waiting time (120 fs). Figure 3a shows the full 2DEV spectrum at 120 fs. In the spectral slices and the spectrum, positive (red in Figure 3a) and negative (blue in Figure 3a) features are ground state bleach (GSB) and excited state absorption (ESA) signals, respectively. To avoid photodamage, the experiments were performed at 77 K (see Section 2.2). The observed IR features correspond to the stretching modes of the 13^1^-keto (1610–1710 cm^–1^) and the 13^3^-ester carbonyl groups (1710–1760 cm^–1^) of Chls *a* and Chls *b*.^17,38,39,55−57^ Both functional groups are good probes of the local protein environment, providing IR markers for the proteins in the PSII-SC. The excitation frequency of 15,200 cm^–1^ marks the separation of two experiments (details in Section 2.2, Figure S1). The lower-frequency excitation range (14,700–15,200 cm^–1^) roughly corresponds to Chl *a* excitation and the higher-frequency excitation range (15,200–15,600 cm^–1^) roughly corresponds to Chl *b* excitation.

In the following, we provide a detailed description of how the 2DEV spectra reveal interprotein EET in the PSII-SC. First, we discuss the assignment of specific IR features. Then, we describe the method used to extract time scales. Finally, we compare the dynamics obtained from 2DEV spectra and the simulation results, which allows us to understand how energy flows between different subunits of the PSII-SC. We want to emphasize that comparisons of 2DEV spectra with visible pump-IR probe spectra are not simple because, in the latter method, there is convolution over the pump spectrum, while the very thing that we rely on in our 2DEV analysis is the ability to spectrally resolve both the excitation and detection axes. The factors that spread out the frequencies on the two axes are uncorrelated and arise from different types of interactions.^34^ In this sense, 2DEV spectroscopy is more similar to heteronuclear 2D NMR spectra (with 10^10^ times higher time resolution) than to either a visible pump-IR probe or 2D electronic spectroscopy. Therefore, in the following, the majority of comparisons were done between the 2DEV spectra of the PSII-SC and its subunits, with occasional comparison with visible pump-IR probe spectra.

The IR structures of PSII-SC at different excitation frequencies in Figure 2a show clear differences. In particular, the overall PSII-SC IR structures at 14,700 and 15,000 cm^–1^ (dark red and red) resemble the PSII-CC IR structures,^39^ whereas the PSII-SC IR structures at 15,300 and 15,600 cm^–1^ (blue and dark blue) resemble those of LHCII.^35^ A further analysis shows that the GSB feature at 1691 cm^–1^ of the PSII-SC at 15,000 cm^–1^ is also present in the PSII-CC at the same excitation frequency, but not in LHCII. While there is currently no 2DEV data on isolated minor antennae CP26 and CP29, we expect them to have similar IR structures to LHCII due to their similar protein structures. Therefore, we assign the feature at 1691 cm^–1^ to the PSII-CC. Furthermore, previous visible pump-IR probe and 2DEV spectroscopy studies on isolated subunits and the PSII-CC have shown that the feature is only found in isolated CP43.^18^ It is absent in the isolated PSII-RC^38,57^ and isolated CP47.^17^ This set of spectra allow us to confidently assign the 1691 cm^–1^ peak exclusively to CP43 at an excitation frequency of 15,000 cm^–1^. Now, we turn the focus to the ESA features in the detection range 1630–1660 cm^–1^ (the green-shaded region in Figure 2). At excitation frequencies of 14,700 and 15,000 cm^–1^, the IR features of the PSII-SC in this detection range are very similar to those of the PSII-CC.^39^ However, at 15,300 and 15,600 cm^–1^, the IR features in this detection range become much more similar to LHCII than the PSII-CC. The features between 1660 and 1680 cm^–1^ are also similar for the PSII-SC and LHCII, and different from those of the PSII-CC. This is expected as these excitation frequencies roughly correspond to the absorption of Chl *b*, present only in the peripheral antennae, and the PSII-CC has only weak absorption at these excitation frequencies. In particular, the strongest feature is observed to be around 1657 cm^–1^ for the PSII-SC and LHCII, which is not a feature for the lower excitation frequency regions of the PSII-SC (14,700 and 15,000 cm^–1^) and for the PSII-CC, suggesting that a strong ESA peak at 1657 cm^–1^ is a marker for the peripheral antennae upon the excitation of 15,300 and 15,600 cm^–1^. Therefore, in the following analyses, we focus on the GSB at 1691 cm^–1^ for the lower excitation frequency range and the ESA at 1657 cm^–1^ for the higher excitation frequency range.

Next, we discuss the extraction of time scales from the dynamics of complex systems, which requires extra care for both experiment and simulation. First, prior assumptions are not desired. Experimentally, traces that contain convoluted dynamics can often be fitted by simple models, and different models can produce equally good fits.^32^ Due to this reason, lifetime density analysis (LDA) is applied to visualize the evolution in the 2DEV spectra and the simulated evolution of the excitation population in each protein (see Section 2.3). Unlike other techniques typically applied for this purpose, such as global and target analysis, LDA does not rely on initial assumptions. Instead, LDA approximates dynamic evolution with hundreds of exponential components to retrieve the amplitude of each lifetime component, leading to bias-free analyses.^43,45^ LDA also provides a way to simultaneously process large parallel data sets, particularly for time-resolved 2D spectra.

The interpretation of the time scales obtained from LDA also needs to be treated carefully. As mentioned, the experimentally obtained traces contain convoluted dynamics. Simulation can generate models that contain microscopic transfer rates, but individual rates cannot represent the overall energy flow. The excitation population evolution depends on all of the microscopic rates. Therefore, the time scales of energy flow obtained from experimental traces and simulated excitation population evolution should represent overall ensemble behavior.

**Figure 4:**
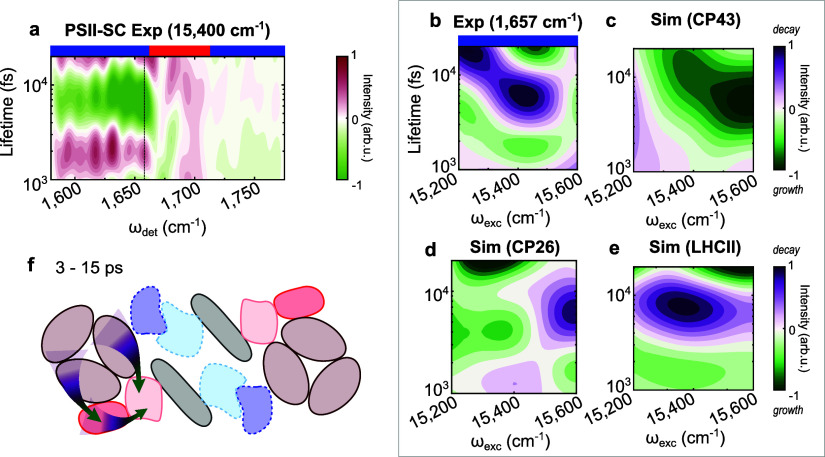
(a) LDM from the 2DEV data averaged over 15,300–15,500 cm^–1^: vertical dashed lines at 1657 cm^–1^; blue (red) bars on top refer to ESA (GSB) signatures. Excitation-dependent LDM of (b) the ESA at 1657 cm^–1^ (reversed in sign) and simulated population evolution of (c) CP43, (d) CP26 and (e) LHCII. Positive amplitudes (purple) indicate decay; negative amplitudes (green) indicate growth. (f) A schematic of the corresponding interprotein EET at 3–15 ps.

For different analysis purposes, we use two different types of lifetime density maps (LDMs) to show the pre-exponential factors, i.e., the amplitudes of each exponential component. First, lifetime vs detection frequency maps (Figures 3e and 4a, pink-green maps) are used to identify the principal lifetime components as well as the IR features involved in the main spectral evolution in each excitation frequency range. However, the fact that 2DEV spectra have positive (GSB) and negative (ESA) features complicates the interpretation of the LDMs. For example, for ESA features, which have a negative sign, decays have negative amplitudes. This is in contrast to GSB features (positive sign), for which decays have positive amplitudes (Figure 3b–d). Therefore, for GSB features, positive and negative LDM amplitudes indicate decay and growth, respectively. In contrast, for ESA features, positive and negative LDM amplitudes indicate growth and decay, respectively. For this reason, the LDM in Figure 4b for the ESA feature at 1657 cm^–1^ is shown with a reverse sign so that, in Figures 3f–i and 4b–e, *all* positive LDM amplitudes represent decays and *all* negative LDM amplitudes represent growths, as specified in the color bars. These are the second type of LDM, lifetime vs excitation frequency maps (purple-green maps), which are used to show the evolution of selected IR features reflecting protein-specific dynamics (independently of the signs of the features, i.e., GSB or ESA), as well as the simulated evolution of the excitation population in the corresponding protein.

Figure 3e shows the LDM for the IR frequencies 1580–1770 cm^–1^ averaged over excitation frequency 14,800–15,200 cm^–1^. Several IR features decay at 0.2–1 ps and 2–10 ps, including the GSB at 1691 cm^–1^, which is a marker of CP43 as discussed above. The LDM of this GSB signature (Figure 3f) agrees extremely well with the decay observed for the simulated evolution of the excitation population in CP43 (Figure 3g). Interestingly, it is clear that the simulated LDMs of CP26 and LHCII (Figure 3h,i) show growths on a similar time scale to the experimental and simulated CP43 decays. This indicates that interprotein EET occurs from CP43 to CP26 and LHCII in 0.2–1 ps and in 2–10 ps (Figure 3j). It is important to note that, while the simulation only focuses on interprotein dynamics, the subps decay of CP43 observed experimentally may also have a contribution from intraprotein EET within CP43, as observed in the isolated PSII-CC.^39^ However, the intraprotein EET in CP43 occurs on a slightly shorter time scale (∼180 fs) than the observed time scale (0.2–1 ps) which suggests that most contribution comes from interprotein EET from CP43 to CP26 and LHCII. Overall, the two time scale ranges observed for the CP43 to CP26/LHCII transfer are much shorter than the reported values for the EET from the core antennae to the RC (which range from 10 to 50 ps in different studies^19,25,29,39,58^).

Figure 4a shows the experimental LDM averaged over excitation frequency 15,300–15,500 cm^–1^ for the PSII-SC, highlighting two main dynamics: EET around 1–3 and 3–15 ps. A wide range of detection frequencies show the evolution at these time scales, including 1657 cm^–1^, which is a marker with the peripheral antennae. Specifically, as there are more Chls in LHCII than in the minor antennae, we expect the ESA feature at 1657 cm^–1^ to originate mostly from LHCII. The LDM associated with the ESA at 1657 cm^–1^ is shown in Figure 4b. For comparison, the LDMs of the simulated evolution for CP43, CP26, and LHCII are shown in Figure 4c–e. It is clear that the evolution of the ESA signature shows strong agreement with the simulated evolution of LHCII, further strengthening our assignment of the ESA at 1657 cm^–1^ to the major antenna. More complex patterns are observed in this excitation frequency range (15,200–15,600 cm^–1^), where mostly Chls *b* (found only in the external antennae) are excited. First, the excitation population in LHCII grows within 1–3 ps, while the only excitation population decay observable within the same time scale is found in CP26, indicating interprotein EET from CP26 to LHCII. In the lifetime range of 3–15 ps, an excitation population growth is observed in CP43 while the excitation population decays in LHCII, indicating EET from LHCII to CP43. Within the same time scale, the dynamics involving CP26 are more complex, showing an excitation frequency dependence. With 15,200–15,400 cm^–1^ excitation, the CP26 LDM shows growth, and the only corresponding decay is found in LHCII, suggesting EET from LHCII to CP26. In the 15,400–15,600 cm^–1^ region, the CP26 LDM shows instead a decay, with the only corresponding growth found in CP43, suggesting EET from CP26 to CP43. Overall, around 1–3 ps, energy flows from CP26 to LHCII. Around 3–15 ps, both CP26 (15,400–15,600 cm^–1^) and LHCII (15,200–15,600 cm^–1^) transfer energy to CP43 (Figure 4f).

In summary, 2DEV spectroscopy combined with the kinetic model constructed by the structure-based simulation shows that excitation of Chls *a* leads to the initial EET from the core to the external antennae, while excitation of Chls *b* is followed by EET from the external antennae to the core, with additional interprotein EET pathways between CP26 and LHCII. In the following, we provide a more detailed discussion of the kinetic model proposed in this work.

### 3.3 Detailed Analysis of the Kinetic Model

**Figure 5:**
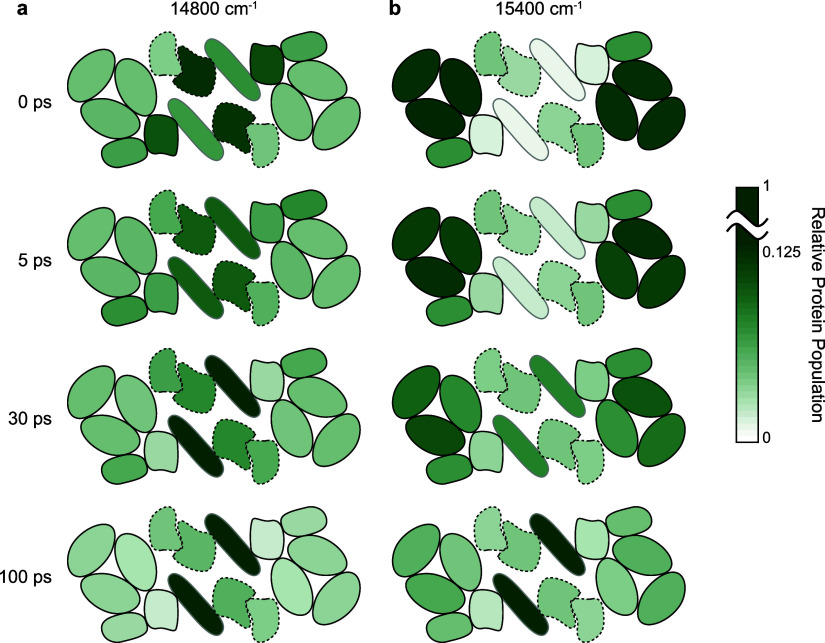
Simulated excitation population in each protein (normalized to the total population) at 0, 5, 30, and 100 ps for excitation frequencies of (a) 14,800 cm^–1^ and (b) 15,400 cm^–1^. The arrangement of the protein subunits is labeled in Figure 1b. The protein subunits on the D1 side are represented by solid lines, and those on the D2 side are represented by dashed lines. The green scale indicates the relative excitation population in each protein (with the maximum on the color scale being 12.5% of the total population to provide visual enhancement for the difference).

2DEV spectroscopy raises the prospect of the extraction of interprotein EET dynamics experimentally. However, the large number of Chls in PSII-SC still causes significant spectral congestion, obscuring the excitation population evolution of certain proteins. For example, the evolution of the peripheral antennae is not observed in the lower-frequency excitation range (14,700–15,200 cm^–1^) of the 2DEV spectra. Instead, the spectra are dominated by the evolution of CP43. This is because the excitation population in a protein needs to undergo enough evolution for the dynamics to be extracted experimentally. To demonstrate this, we simulated the evolution of the excitation population in each protein subunit. Figure 5 shows the simulated energy distribution at different time delays in the PSII-SC at two selected excitation frequencies: 14,800 cm^–1^, representing Chls *a* excitation (Figure 5a), and 15,400 cm^–1^, representing Chls *b* excitation (Figure 5b). We note that the color scale is set to be maximized at 12.5% to visually enhance the population evolution. For example, at 5 ps upon excitation at 14,800 cm^–1^, the growth of RC may seem surprising considering the time scales for the transfer from core antennae to the RC. However, the actual calculated growth is around 3%, which is expected for 5 ps taking into account the mean fluorescence lifetime of about 150 ps^25^ for the C_2_S_2_-type PSII-SC. The initial excitation distributions (0 ps) at the two excitation frequencies were obtained based on the simulated absorption spectra of all pigments (see Section 2.4 for details). The excitation distribution evolution clearly shows that the challenges of extracting interprotein EET in the PSII-SC from the experiment originate not only from the significant spectral congestion but also from the intrinsic dynamics of the system. At 14,800 cm^–1^, simulations show that only the core antennae undergo obvious population evolution while being already partially excited, the population in the peripheral antennae barely changes. Therefore, it is expected that CP43 signatures on the 2DEV spectra show more evolution compared to the peripheral antennae in this excitation frequency range. Similarly, the simulated evolution of the excitation population shows that LHCII changes the most upon excitation at 15,400 cm^–1^, supporting the experimental observation that LHCII signatures show more dynamical evolution on the 2DEV spectra in this excitation frequency range and obscure the dynamics of the other protein subunits. These predictions made by the structure-based simulation completely match the observation from the 2DEV experiments, showing that the nature of the dynamic evolution can pose an additional constraint for experimental analyses, particularly in complex systems such as the PSII-SC.

**Figure 6:**
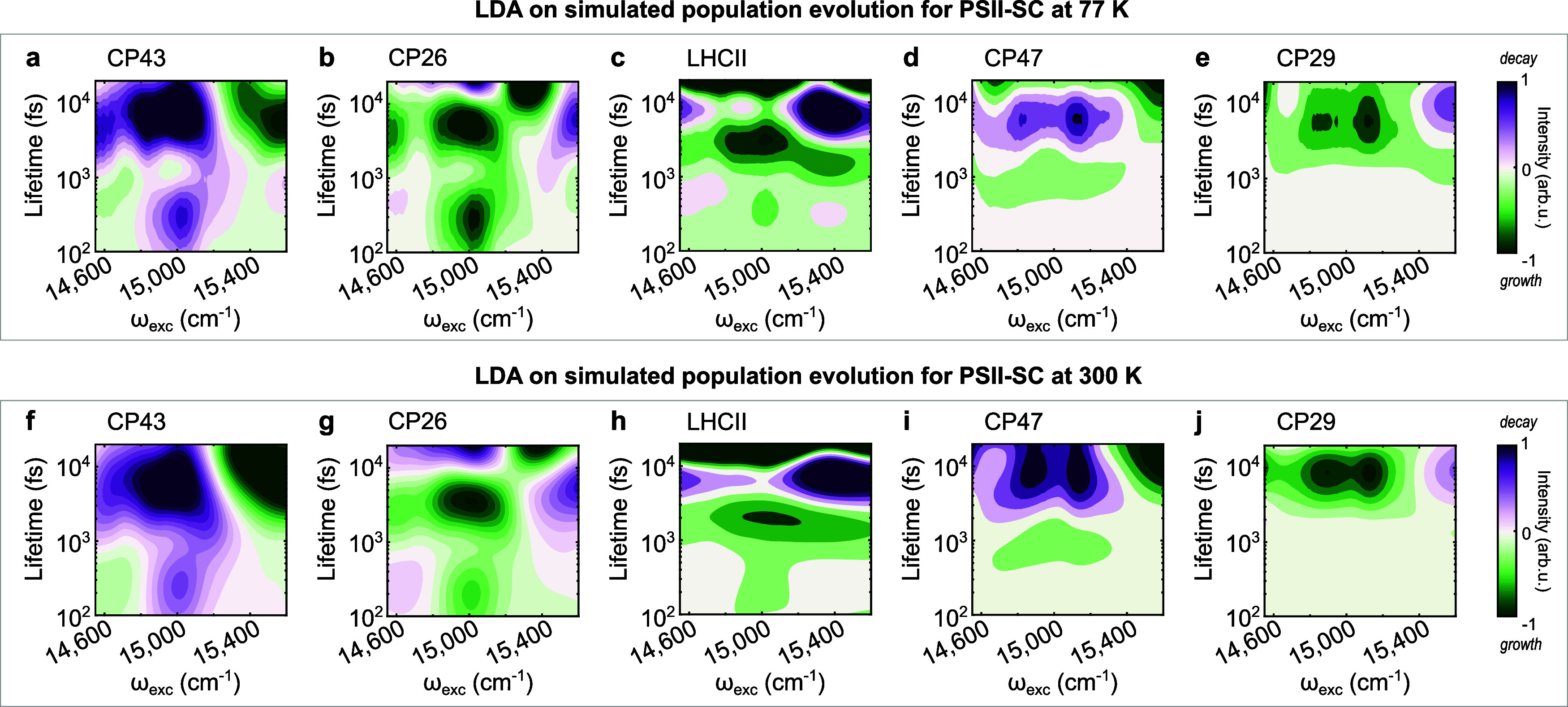
Simulated PSII-SC excitation-dependent LDM of (a) CP43, (b) CP26, (c) LHCII, (d) CP47 and (e) CP29 at 300 K for all of the excitation frequency ranges discussed. Simulated PSII-SC excitation-dependent LDM of (f) CP43, (g) CP26, (h) LHCII, (i) CP47 and (j) CP29 at 300 K for all the excitation frequency ranges discussed. Positive amplitudes (purple) indicate decay; negative amplitudes (green) indicate growth.

Another important factor that needs to be addressed is that only the subunits with detectable IR signatures can be tracked experimentally via 2DEV spectroscopy. This limits the amount of information retrievable for CP47, which has been shown to have weaker IR features than CP43 in the 2DEV spectra of the PSII-CC.^39^ To understand the EET dynamics on the D2 side, at present, we can rely on simulations that show great consistency with the experimental results for the dynamics on the D1 side. Figure 6a–j shows the LDM for the simulated population evolution of each protein in the PSII-SC at all excitation frequencies discussed (14,600–15,600 cm^–1^). The kinetic model shows that the EET directions of the proteins along the D2 branch, CP47 and CP29 (Figure 6d,e), are similar to those of the corresponding D1 proteins, CP43 and CP26 (Figure 6a,b, respectively). The excitation of Chls *a* leads to interprotein EET from CP47 to CP29 while the excitation of Chls *b* shows EET from CP29 to CP47, consistent with the excitation frequency-dependent EET directions between the core and peripheral antennae observed for the D1 side. Both processes take place in 3–10 ps. The most significant difference is the 0.2–1 ps interprotein EET between CP43 and LCHII/CP26 observed in the D1 proteins in the lower-frequency excitation range, which is not observed for the proteins on the D2 side.

Finally, the experiments were performed at 77 K to avoid photodamage. This raises a question about the applicability of the experimental data and the conclusions we can draw from them about natural photosynthesis. To understand the effect of the temperature, simulations of the dynamics at 300 K were also performed. The model shows that the dynamics at 77 K (Figure 6a–e) and 300 K (Figure 6f–j) are almost identical within the first 20 ps. This confirms that the experiments performed at cryogenic temperatures can also provide insights relevant to physiological conditions. Another concern for the cryogenic condition of the 2DEV experiment is that the RC stays closed. However, it has been shown that the primary charge separation occurs on a 1.5 and 3.3 ps time scale for open and closed RC, respectively.^59^ Both of these time scales are short compared to the transfer from the core antennae to the RCs. 2DEV experiments on the isolated PSII-RC^60^ and the isolated PSII-CC,^39^ which have open and closed RCs respectively, also reveal similar time scales for charge separation processes. Therefore, we expect the bidirectional energy flow between the peripheral and core antennae within the first 20 ps to have similar dynamics in the presence of open RCs.

### 3.4 Efficiency and Photoprotection

In the previous sections, we showed that in the lower-frequency excitation range the energy quickly leaves the core to explore the peripheral antennae, faster than it is transferred to the RC. The ability of the former process to compete with the latter is crucial for photoprotection. Indeed, it has been shown that photoprotection mostly takes place within the peripheral antennae via interactions with carotenoids.^2,24,52,61^ On the other hand, for the higher-frequency excitation range, in which mostly Chls *b* (found only in the peripheral antennae) are excited, the net EET direction is the opposite. The absorbed energy, mostly distributed in the external antennae upon excitation, has already a higher chance to explore the quenching sites due to their proximity to the initially excited pigments, which leads to effective protection. Under low light conditions, where the quenchers are inactive, the EET pathways quickly guide the unquenched energy from the external antennae toward the core to reach the RC.

One key factor that allows the bidirectionality of the energy flow, which facilitates the switch between efficient and photoprotective mode, is the time scale of different competing EET pathways. In the lower-frequency excitation range, energy is observed to transfer from CP43 to CP26 and LHCII on a sub-ps time scale. This pronounced connection between the core and the peripheral antennae on the D1 side is a result of the short center-to-center distances between certain pairs of Chls of CP26/LHCII and CP43, e.g., C601–C513 (CP26-CP43: 12.6Å), C614–C503 (CP26-CP43: 16.02 Å), and C611–C506 (LHCII-CP43: 17.05 Å).^13^ This kind of design allows the EET pathways that guide energy out of the core to compete with EET from CP43 to the RC, which has been shown to happen on a time scale of 10 s of ps.^19,25,29,39,58^ This indicates that excitations in CP43 have a much higher chance to move to CP26 and LHCII than to directly enter the RC. Interestingly, this allows CP26 to play an important role in the overall EET network despite being located on the periphery of the PSII-SC, showing the importance of investigating the dynamics in the complete PSII-SC to elucidate its design principles. Sub-ps EET between different subunits was not observed in the simulated population evolution of the subunits along the D2 side (Figure 6d,e,i,j). However, within 3–10 ps, EET from the core to the peripheral antennae is observed along both sides. This still allows EET from CP47 to CP29 to compete with EET from CP47 to the RC as the latter has been shown to be slower compared to EET from CP43 to the RC.^29^ On the other hand, in the higher-frequency excitation range where excitation is further away from the RC, interprotein EET to the core antennae still occurs in 3–15 ps. This is a similar time scale to the EET from the core antenna to the RC, which guarantees that energy reaches the RC before it is dissipated.

**Figure 7:**
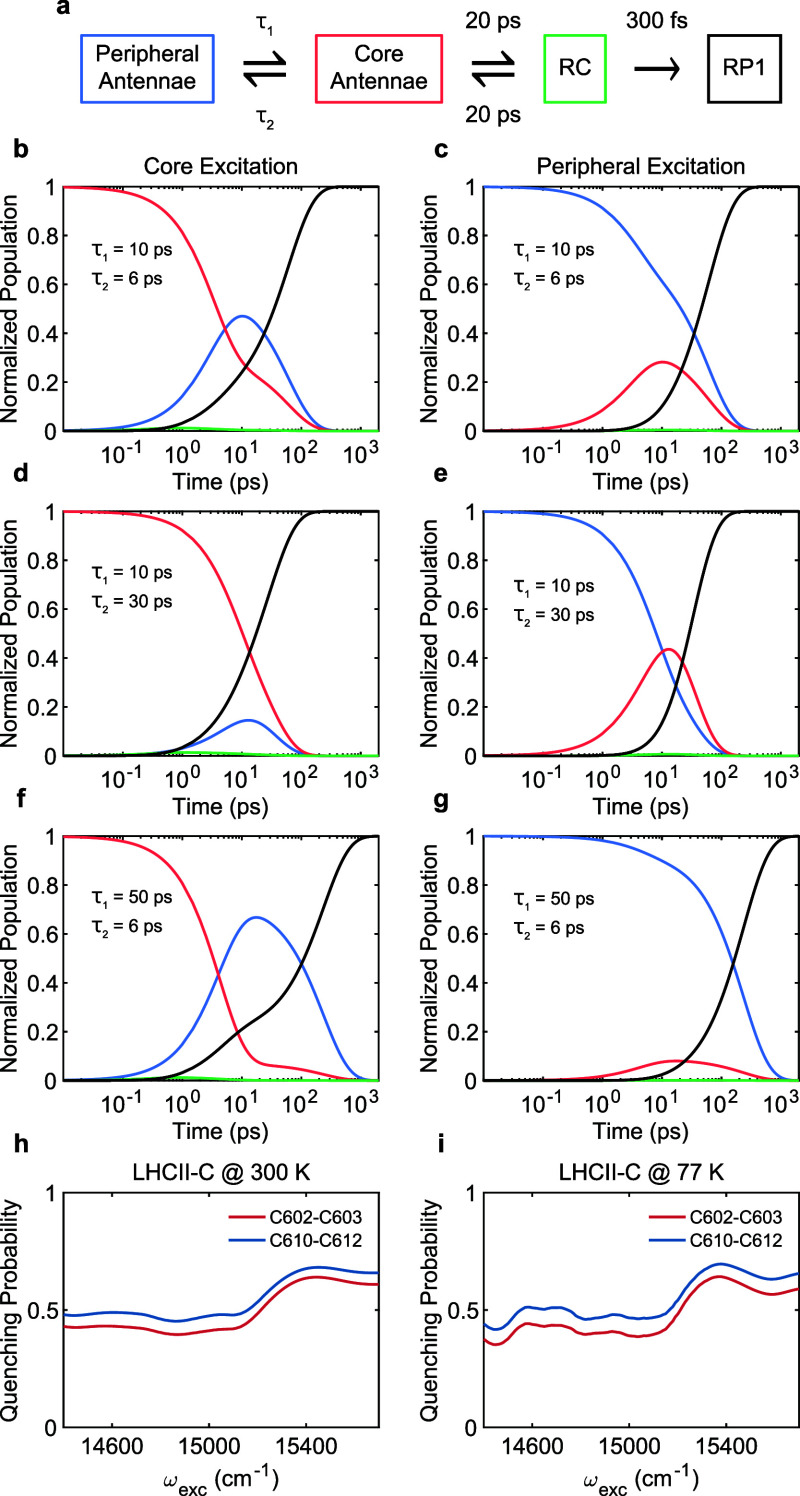
(a) Coarse-grained kinetic model with the time scales approximated from the experiment and literature.^19,25,29,39,58^ RP1 is the primary charge transfer state. Population evolution upon the excitation of core/peripheral antennae, respectively, when the time scales are (b, c) extracted from the 2DEV measurement (d, e) 5-fold slower for the core to peripheral antennae transfer and (f, g) 5-fold slower for the peripheral to core antennae transfer. The color scheme of (b–g) is consistent with those of the compartments in (a). Simulated excitation-dependent quenching probability when active quenchers are placed in the LHCII-C monomer (see Figure 1) for (h), 300 K and (i), 77 K. The quenchers are placed in the proximity of C602–C603 (red) and C610–C612 clusters (blue). A detailed description can be found in Figure S3 and in Section 2.4.

To illustrate the significance of the actual time scales of energy flow in the PSII-SC, we make use of a conceptual coarse-grained model (Figure 7a). By employing the time scales observed in our experiments in the coarse-grained model, we demonstrate how the balance between efficient charge separation and photoprotection relies on the EET time scales between peripheral and core antennae. In the coarse-grained model, we only focus on the few picosecond time scales, observed for the antennae complexes on both D1 and D2 sides, instead of the subpicosecond transfer present only between CP43 and CP26/LHCII. Figure 7b,c shows that under natural operation conditions of the PSII-SC, the population of the peripheral antennae (blue) grows faster than that of the charge transfer state (black), allowing energy to visit the quenching sites before entering the RC. A 5-fold slower EET from the core antennae to the peripheral antennae (Figure 7d,e) would greatly reduce the probability of visiting the quenching sites because the transfer to the RC (black) would become dominant as the population of the peripheral antennae (blue) is suppressed. This would limit the ability of the PSII-SC to quench excessive excitation under high-light conditions. On the other hand, a 5-fold slower transfer from the peripheral to the core antennae (Figure 7f,g) would decrease the photosynthetic efficiency in low-light conditions as energy cannot fully reach the charge transfer state (black) before undergoing dissipative pathways. Such a balance shows that the structural arrangement of the peripheral and the core antennae allows the PSII-SC to work in a regime where EET occurs on time scales optimized for both efficiency and photoprotection. Noticeably, the time scales found in this work are designed to be balanced with the EET time scale from the core antennae to the RC, which is limited by the large distance required for protecting the separated charges.^62^ This applies for both D1 and D2 sides, as both sides show transfer between core and peripheral antennae on a few-ps time scales. Furthermore, the effect of transfer time scales from the peripheral to the core antennae shows that there is an upper limit for the antenna size (larger than the C_2_S_2_-type PSII-SC), as proposed by Croce and co-workers.^63^ An increased antenna size leads to an increased overall absorption cross-section, which is necessary under low-light conditions. However, it also leads to slower EET to PSII-CC, imposing an upper limit on the antenna size for optimal photosynthetic efficiency.

Overall, the kinetic network in the PSII-SC is designed so that regardless of where excitation is in the PSII-SC, the excitation energy has a high chance of visiting the quenching sites. There, it can either be quenched in high-light conditions or continue to be transferred to the RC well before nonradiative losses occur in low-light conditions. Figure 7h,i (and Figure S3) shows that, by placing the quencher in the peripheral antenna (e.g., LHCII), the protection ability of the quencher does not depend on the excitation axis and therefore does not depend on the excitation location. This shows that nonphotochemical quenching, which uses a feedback loop to activate/deactivate quenching depending on the light intensity,^2^ can only work in combination with the bidirectional EET pathways with balanced time scales. In other words, a fine kinetic balance is necessary to allow effective photoprotection under high-light conditions (quenching activated) while guaranteeing photosynthetic efficiency under low-light conditions (quenching inactive). How the balance (Figure 7b,c) is achieved depends on the detailed microscopic rates of energy flow between the subunits of the PSII-SC that are obtained by the comparison of the experimental and simulation data described above.

## 4 Conclusions

It has been known for several decades that the PSII-SC has a rather flat energy landscape,^3−7^ in contrast to the energy funnel that other photosynthetic systems exhibit.^1^ While it is speculated that the flat energy landscape (or shallow funnel) design is related to photoprotection,^4,7^ the exact working mechanism cannot be easily inferred without a deeper understanding of the EET dynamics in the PSII-SC.

In this work, we reveal the connection between the EET network and the functions of the C_2_S_2_-type PSII-SC. We show that energy can flow out of the PSII-CC and transfer from the peripheral antenna system back into the core, increasing the probability of visiting the quenching sites before entering the RC. The time scales for the net EET between peripheral and core antennae, controlled by the microscopic transfer rates, are finely balanced to facilitate the bi-directionality of energy flow in the PSII-SC. It is reasonable to argue that such an optimized kinetic network is made possible by the rather flat energy landscape within the multicomponent structure of the PSII-SC. Ultimately, the strategy to have bidirectional energy transfer pathways enables switching between efficient energy conversion and effective photoprotection, responding to the fluctuating sunlight intensity.

Understanding the functional mechanism of the PSII-SC can allow us to improve the bioinspired design of solar energy devices, enabling fine control of the solar energy conversion processes. Additionally, the optimization of the response of crop plants to fluctuating light levels has emerged as a major step in the improvement of crop yield.^10^ As the location and time scales associated with nonphotochemical quenching are elucidated, the detailed understanding of energy flow pathways and time scales, as well as the response to differing solar wavelengths, will aid in formulating the strategies to continue to enhance the yields of food crops, as is necessary over the next 20–30 years.

## Supporting Information Available

Simulation parameters and data analysis details (PDF)

## References

[ref1] Blankenship R. E. Molecular mechanisms of photosynthesis; John Wiley & Sons, 2021.

[ref2] Demmig-Adams B.; Garab G.; Adams W. III; Govindjee U. Non-photochemical quenching and energy dissipation in plants, algae and cyanobacteria; Springer, 2014; 40.

[ref3] Jennings R. C.; Bassi R.; Garlaschi F. M.; Dainese P.; Zucchelli G. Distribution of the chlorophyll spectral forms in the chlorophyll-protein complexes of photosystem II antenna. Biochemistry 1993, 32, 3203–3210. 10.1021/bi00064a002. 8461288

[ref4] Jennings R. C.; Garlaschi F. M.; Bassi R.; Zucchelli G.; Vianelli A.; Dainese P. A study of photosystem II fluorescence emission in terms of the antenna chlorophyll-protein complexes. Biochimica et Biophysica Acta (BBA)-Bioenergetics 1993, 1183, 194–200. 10.1016/0005-2728(93)90018-B.

[ref5] Shibata Y.; Nishi S.; Kawakami K.; Shen J.-R.; Renger T. Photosystem II does not possess a simple excitation energy funnel: time-resolved fluorescence spectroscopy meets theory. J. Am. Chem. Soc. 2013, 135, 6903–6914. 10.1021/ja312586p. 23537277 PMC3650659

[ref6] Croce R.; Van Amerongen H. Natural strategies for photosynthetic light harvesting. Nat. Chem. Biol. 2014, 10, 492–501. 10.1038/nchembio.1555. 24937067

[ref7] Kreisbeck C.; Aspuru-Guzik A. Efficiency of energy funneling in the photosystem II supercomplex of higher plants. Chemical Science 2016, 7, 4174–4183. 10.1039/C5SC04296H. 30155062 PMC6014079

[ref8] Oliver T. A.; Lewis N. H.; Fleming G. R. Correlating the motion of electrons and nuclei with two-dimensional electronic–vibrational spectroscopy. Proc. Natl. Acad. Sci. U. S. A. 2014, 111, 10061–10066. 10.1073/pnas.1409207111. 24927586 PMC4104903

[ref9] Lewis N. H. C.; Dong H.; Oliver T. A. A.; Fleming G. R. Measuring correlated electronic and vibrational spectral dynamics using line shapes in two-dimensional electronic-vibrational spectroscopy. J. Chem. Phys. 2015, 142, 174202 10.1063/1.4919686. 25956093

[ref10] De Souza A. P.; Burgess S. J.; Doran L.; Hansen J.; Manukyan L.; Maryn N.; Gotarkar D.; Leonelli L.; Niyogi K. K.; Long S. P. Soybean photosynthesis and crop yield are improved by accelerating recovery from photoprotection. Science 2022, 377, 851–854. 10.1126/science.adc9831. 35981033

[ref11] Albanese P.; Manfredi M.; Meneghesso A.; Marengo E.; Saracco G.; Barber J.; Morosinotto T.; Pagliano C. Dynamic reorganization of photosystem II supercomplexes in response to variations in light intensities. Biochimica et Biophysica Acta (BBA)-Bioenergetics 2016, 1857, 1651–1660. 10.1016/j.bbabio.2016.06.011. 27378191

[ref12] Croce R.; van Amerongen H. Light harvesting in oxygenic photosynthesis: Structural biology meets spectroscopy. Science 2020, 369, eaay2058 10.1126/science.aay2058. 32820091

[ref13] Wei X.; Su X.; Cao P.; Liu X.; Chang W.; Li M.; Zhang X.; Liu Z. Structure of spinach photosystem II–LHCII supercomplex at 3.2 Å resolution. Nature 2016, 534, 69–74. 10.1038/nature18020. 27251276

[ref14] Steffen M. A.; Lao K.; Boxer S. G. Dielectric asymmetry in the photosynthetic reaction center. Science 1994, 264, 810–816. 10.1126/science.264.5160.810. 17794722

[ref15] Diner B. A.; Rappaport F. Structure, dynamics, and energetics of the primary photochemistry of photosystem II of oxygenic photosynthesis. Annual review of plant biology 2002, 53, 551–580. 10.1146/annurev.arplant.53.100301.135238. 12221988

[ref16] Salverda J. M.; Vengris M.; Krueger B. P.; Scholes G. D.; Czarnoleski A. R.; Novoderezhkin V.; Van Amerongen H.; Van Grondelle R. Energy transfer in light-harvesting complexes LHCII and CP29 of spinach studied with three pulse echo peak shift and transient grating. Biophys. J. 2003, 84, 450–465. 10.1016/s0006-3495(03)74865-6. 12524298 PMC1302626

[ref17] Groot M. L.; Breton J.; van Wilderen L. J.; Dekker J. P.; van Grondelle R. Femtosecond visible/visible and visible/mid-IR pump- probe study of the photosystem II core antenna complex CP47. J. Phys. Chem. B 2004, 108, 8001–8006. 10.1021/jp037966s. 17550278

[ref18] Di Donato M.; van Grondelle R.; van Stokkum I. H.; Groot M. L. Excitation energy transfer in the photosystem II core antenna complex CP43 studied by femtosecond visible/visible and visible/mid-infrared pump probe spectroscopy. J. Phys. Chem. B 2007, 111, 7345–7352. 10.1021/jp068315+. 17550278

[ref19] Pawlowicz N. P.; Groot M.-L.; Van Stokkum I.; Breton J.; van Grondelle R. Charge separation and energy transfer in the photosystem II core complex studied by femtosecond midinfrared spectroscopy. Biophys. J. 2007, 93, 2732–2742. 10.1529/biophysj.107.105452. 17573421 PMC1989691

[ref20] Schlau-Cohen G. S.; Calhoun T. R.; Ginsberg N. S.; Read E. L.; Ballottari M.; Bassi R.; van Grondelle R.; Fleming G. R. Pathways of energy flow in LHCII from two-dimensional electronic spectroscopy. J. Phys. Chem. B 2009, 113, 15352–15363. 10.1021/jp9066586. 19856954

[ref21] Marin A.; Passarini F.; Croce R.; Van Grondelle R. Energy transfer pathways in the CP24 and CP26 antenna complexes of higher plant photosystem II: a comparative study. Biophysical journal 2010, 99, 4056–4065. 10.1016/j.bpj.2010.10.034. 21156149 PMC3000508

[ref22] Pan J.; Gelzinis A.; Chorošajev V.; Vengris M.; Senlik S. S.; Shen J.-R.; Valkunas L.; Abramavicius D.; Ogilvie J. P. Ultrafast energy transfer within the photosystem II core complex. Phys. Chem. Chem. Phys. 2017, 19, 15356–15367. 10.1039/C7CP01673E. 28574545

[ref23] Do T. N.; Nguyen H. L.; Akhtar P.; Zhong K.; Jansen T. L.; Knoester J.; Caffarri S.; Lambrev P. H.; Tan H.-S. Ultrafast excitation energy transfer dynamics in the LHCII–CP29–CP24 subdomain of plant photosystem II. J. Phys. Chem. Lett. 2022, 13, 4263–4271. 10.1021/acs.jpclett.2c00194. 35522529

[ref24] Van Oort B.; Alberts M.; De Bianchi S.; DallOsto L.; Bassi R.; Trinkunas G.; Croce R.; Van Amerongen H. Effect of antenna-depletion in Photosystem II on excitation energy transfer in Arabidopsis thaliana. Biophys. J. 2010, 98, 922–931. 10.1016/j.bpj.2009.11.012. 20197046 PMC2830445

[ref25] Caffarri S.; Broess K.; Croce R.; van Amerongen H. Excitation energy transfer and trapping in higher plant photosystem II complexes with different antenna sizes. Biophysical journal 2011, 100, 2094–2103. 10.1016/j.bpj.2011.03.049. 21539776 PMC3149253

[ref26] Miloslavina Y.; de Bianchi S.; DallOsto L.; Bassi R.; Holzwarth A. R. Quenching in Arabidopsis thaliana mutants lacking monomeric antenna proteins of photosystem II. J. Biol. Chem. 2011, 286, 36830–36840. 10.1074/jbc.M111.273227. 21844190 PMC3196121

[ref27] Broess K.; Trinkunas G.; van Hoek A.; Croce R.; van Amerongen H. Determination of the excitation migration time in photosystem II: Consequences for the membrane organization and charge separation parameters. Biochimica et Biophysica Acta (BBA)-Bioenergetics 2008, 1777, 404–409. 10.1016/j.bbabio.2008.02.003. 18355436

[ref28] Broess K.; Trinkunas G.; Van Der Weij-De C. D.; Dekker J. P.; van Hoek A.; van Amerongen H. Excitation energy transfer and charge separation in photosystem II membranes revisited. Biophys. J. 2006, 91, 3776–3786. 10.1529/biophysj.106.085068. 16861268 PMC1630486

[ref29] Raszewski G.; Renger T. Light harvesting in photosystem II core complexes is limited by the transfer to the trap: can the core complex turn into a photoprotective mode?. J. Am. Chem. Soc. 2008, 130, 4431–4446. 10.1021/ja7099826. 18327941

[ref30] Novoderezhkin V.; Marin A.; van Grondelle R. Intra-and inter-monomeric transfers in the light harvesting LHCII complex: the Redfield–Förster picture. Phys. Chem. Chem. Phys. 2011, 13, 17093–17103. 10.1039/c1cp21079c. 21866281

[ref31] Mascoli V.; Novoderezhkin V.; Liguori N.; Xu P.; Croce R. Design principles of solar light harvesting in plants: Functional architecture of the monomeric antenna CP29. Biochimica Et Biophysica Acta (BBA)-Bioenergetics 2020, 1861, 148156 10.1016/j.bbabio.2020.148156. 31987813

[ref32] Bennett D. I.; Amarnath K.; Fleming G. R. A structure-based model of energy transfer reveals the principles of light harvesting in photosystem II supercomplexes. J. Am. Chem. Soc. 2013, 135, 9164–9173. 10.1021/ja403685a. 23679235

[ref33] Chmeliov J.; Trinkunas G.; van Amerongen H.; Valkunas L. Light harvesting in a fluctuating antenna. J. Am. Chem. Soc. 2014, 136, 8963–8972. 10.1021/ja5027858. 24870124

[ref34] Arsenault E. A.; Bhattacharyya P.; Yoneda Y.; Fleming G. R. Two-dimensional electronic–vibrational spectroscopy: Exploring the interplay of electrons and nuclei in excited state molecular dynamics. J. Chem. Phys. 2021, 155, 020901 10.1063/5.0053042. 34266264

[ref35] Lewis N. H.; Gruenke N. L.; Oliver T. A.; Ballottari M.; Bassi R.; Fleming G. R. Observation of electronic excitation transfer through light harvesting complex II using two-dimensional electronic–vibrational spectroscopy. journal of physical chemistry letters 2016, 7, 4197–4206. 10.1021/acs.jpclett.6b02280. 27704843 PMC6314458

[ref36] Arsenault E. A.; Yoneda Y.; Iwai M.; Niyogi K. K.; Fleming G. R. Vibronic mixing enables ultrafast energy flow in light-harvesting complex II. Nat. Commun. 2020, 11, 1460 10.1038/s41467-020-14970-1. 32193383 PMC7081214

[ref37] Arsenault E. A.; Yoneda Y.; Iwai M.; Niyogi K. K.; Fleming G. R. The role of mixed vibronic Qy-Qx states in green light absorption of light-harvesting complex II. Nat. Commun. 2020, 11, 6011 10.1038/s41467-020-19800-y. 33243997 PMC7691517

[ref38] Yoneda Y.; Arsenault E. A.; Yang S.-J.; Orcutt K.; Iwai M.; Fleming G. R. The initial charge separation step in oxygenic photosynthesis. Nat. Commun. 2022, 13, 2275 10.1038/s41467-022-29983-1. 35477708 PMC9046298

[ref39] Yang S. J.; Arsenault E. A.; Orcutt K.; Iwai M.; Yoneda Y.; Fleming G. R. From antenna to reaction center: Pathways of ultrafast energy and charge transfer in photosystem II. Proc. Natl. Acad. Sci. U. S. A. 2022, 119, e2208033119 10.1073/pnas.2208033119. 36215463 PMC9586314

[ref40] Nguyen H. H.; Song Y.; Maret E. L.; Silori Y.; Willow R.; Yocum C. F.; Ogilvie J. P. Charge separation in the photosystem II reaction center resolved by multispectral two-dimensional electronic spectroscopy. Sci. Adv. 2023, 9, eade7190 10.1126/sciadv.ade7190. 37134172 PMC10156117

[ref41] Berthold D. A.; Babcock G. T.; Yocum C. F. A highly resolved, oxygen-evolving photosystem II preparation from spinach thylakoid membranes. FEBS letters 1981, 134, 231–234. 10.1016/0014-5793(81)80608-4.

[ref42] Caffarri S.; Kouřil R.; Kereïche S.; Boekema E. J.; Croce R. Functional architecture of higher plant photosystem II supercomplexes. EMBO journal 2009, 28, 3052–3063. 10.1038/emboj.2009.232. 19696744 PMC2760109

[ref43] Slavov C.; Hartmann H.; Wachtveitl J. Implementation and evaluation of data analysis strategies for time-resolved optical spectroscopy. Analytical chemistry 2015, 87, 2328–2336. 10.1021/ac504348h. 25590674

[ref44] van Stokkum I. H.; Larsen D. S.; Van Grondelle R. Global and target analysis of time-resolved spectra. Biochimica et Biophysica Acta (BBA)-Bioenergetics 2004, 1657, 82–104. 10.1016/j.bbabio.2004.04.011. 15238266

[ref45] Dorlhiac G. F.; Fare C.; van Thor J. J. PyLDM-An open source package for lifetime density analysis of time-resolved spectroscopic data. PLoS computational biology 2017, 13, e1005528 10.1371/journal.pcbi.1005528. 28531219 PMC5460884

[ref46] Madjet M.; Abdurahman A.; Renger T. Intermolecular Coulomb couplings from ab initio electrostatic potentials: application to optical transitions of strongly coupled pigments in photosynthetic antennae and reaction centers. J. Phys. Chem. B 2006, 110, 17268–17281. 10.1021/jp0615398. 16928026

[ref47] Renger T.; Trostmann I.; Theiss C.; Madjet M.; Richter M.; Paulsen H.; Eichler H.; Knorr A.; Renger G. Refinement of a structural model of a pigment- protein complex by accurate optical line shape theory and experiments. J. Phys. Chem. B 2007, 111, 10487–10501. 10.1021/jp0717241. 17696386

[ref48] Raszewski G.; Diner B. A.; Schlodder E.; Renger T. Spectroscopic properties of reaction center pigments in photosystem II core complexes: revision of the multimer model. Biophysical journal 2008, 95, 105–119. 10.1529/biophysj.107.123935. 18339736 PMC2426664

[ref49] Olszówka D.; Krawczyk S.; Maksymiec W. A study of molecular interactions in light-harvesting complexes LHCIIb, CP29, CP26 and CP24 by Stark effect spectroscopy. Biochimica et Biophysica Acta (BBA)-Bioenergetics 2004, 1657, 61–70. 10.1016/j.bbabio.2004.04.004. 15238212

[ref50] Raszewski G.; Saenger W.; Renger T. Theory of optical spectra of photosystem II reaction centers: location of the triplet state and the identity of the primary electron donor. Biophys. J. 2005, 88, 986–998. 10.1529/biophysj.104.050294. 15556979 PMC1305170

[ref51] Ballottari M.; Mozzo M.; Girardon J.; Hienerwadel R.; Bassi R. Chlorophyll triplet quenching and photoprotection in the higher plant monomeric antenna protein Lhcb5. J. Phys. Chem. B 2013, 117, 11337–11348. 10.1021/jp402977y. 23786371

[ref52] Ruban A. V.; Berera R.; Ilioaia C.; Van Stokkum I. H.; Kennis J. T.; Pascal A. A.; Van Amerongen H.; Robert B.; Horton P.; Van Grondelle R. Identification of a mechanism of photoprotective energy dissipation in higher plants. Nature 2007, 450, 575–578. 10.1038/nature06262. 18033302

[ref53] Park S.; Fischer A. L.; Steen C. J.; Iwai M.; Morris J. M.; Walla P. J.; Niyogi K. K.; Fleming G. R. Chlorophyll-carotenoid excitation energy transfer in high-light-exposed thylakoid membranes investigated by snapshot transient absorption spectroscopy. J. Am. Chem. Soc. 2018, 140, 11965–11973. 10.1021/jacs.8b04844. 30183270

[ref54] Son M.; Pinnola A.; Gordon S. C.; Bassi R.; Schlau-Cohen G. S. Observation of dissipative chlorophyll-to-carotenoid energy transfer in light-harvesting complex II in membrane nanodiscs. Nat. Commun. 2020, 11, 1295 10.1038/s41467-020-15074-6. 32157079 PMC7064482

[ref55] Nabedryk E.; Andrianambinintsoa S.; Berger G.; Leonhard M.; Mäntele W.; Breton J. Characterization of bonding interactions of the intermediary electron acceptor in the reaction center of photosystem II by FTIR spectroscopy. Biochimica et Biophysica Acta (BBA)-Bioenergetics 1990, 1016, 49–54. 10.1016/0005-2728(90)90005-O.

[ref56] Noguchi T.; Tomo T.; Inoue Y. Fourier transform infrared study of the cation radical of P680 in the photosystem II reaction center: evidence for charge delocalization on the chlorophyll dimer. Biochemistry 1998, 37, 13614–13625. 10.1021/bi9812975. 9753448

[ref57] Groot M. L.; Pawlowicz N. P.; van Wilderen L. J.; Breton J.; van Stokkum I. H.; van Grondelle R. Initial electron donor and acceptor in isolated photosystem II reaction centers identified with femtosecond mid-IR spectroscopy. Proc. Natl. Acad. Sci. U. S. A. 2005, 102, 13087–13092. 10.1073/pnas.0503483102. 16135567 PMC1196200

[ref58] Müh F.; Zouni A. Structural basis of light-harvesting in the photosystem II core complex. Protein Sci. 2020, 29, 1090–1119. 10.1002/pro.3841. 32067287 PMC7184784

[ref59] van der Weij-de Wit C.; Dekker J.; van Grondelle R.; Van Stokkum I. Charge separation is virtually irreversible in photosystem II core complexes with oxidized primary quinone acceptor. J. Phys. Chem. A 2011, 115, 3947–3956. 10.1021/jp1083746. 21341818

[ref60] Yoneda Y.; Mora S. J.; Shee J.; Wadsworth B. L.; Arsenault E. A.; Hait D.; Kodis G.; Gust D.; Moore G. F.; Moore A. L.; et al. Electron–nuclear dynamics accompanying proton-coupled electron transfer. J. Am. Chem. Soc. 2021, 143, 3104–3112. 10.1021/jacs.0c10626. 33601880

[ref61] DallOsto L.; Cazzaniga S.; Bressan M.; Paleček D.; Židek K.; Niyogi K. K.; Fleming G. R.; Zigmantas D.; Bassi R. Two mechanisms for dissipation of excess light in monomeric and trimeric light-harvesting complexes. Nat. Plants 2017, 3, 17033 10.1038/nplants.2017.33. 28394312

[ref62] Vasil’ev S.; Orth P.; Zouni A.; Owens T. G.; Bruce D. Excited-state dynamics in photosystem II: Insights from the x-ray crystal structure. Proc. Natl. Acad. Sci. U. S. A. 2001, 98, 8602–8607. 10.1073/pnas.141239598. 11459991 PMC37482

[ref63] Wientjes E.; van Amerongen H.; Croce R. Quantum yield of charge separation in photosystem II: functional effect of changes in the antenna size upon light acclimation. J. Phys. Chem. B 2013, 117, 11200–11208. 10.1021/jp401663w. 23534376

