# Supplementary material for: Bidirectional Energy Flow in the Photosystem II Supercomplex
Source: J Phys Chem B. 2024 Aug 14;128(33):7941–53. doi: 10.1021/acs.jpcb.4c02508 (PMC11345834; doi:10.1021/acs.jpcb.4c02508)
Supplement: Supplementary file 1 — jp4c02508_si_001.pdf [file jp4c02508_si_001.pdf]

Supporting Information for Bidirectional Energy Flow in the Photosystem II Supercomplex

Cristina Leonardo,[†,‡,@] Shiun-Jr Yang,[¶,‡,§,@] Kaydren Orcutt,[¶,‡,‖] Masakazu Iwai,[⊥,‡] Eric A. Arsenault,[¶,‡,§,#] and Graham R. Fleming[*,¶,‡,§]

†*Department of Chemistry, University of California, Berkeley, CA, 94720, USA*

‡*Molecular Biophysics and Integrated Bioimaging Division, Lawrence Berkeley National Laboratory, Berkeley, CA, 94720, USA*

1 ¶*Department of Chemistry, University of California, Berkeley, Berekeley, CA, 94720, USA*

§*Kavli Energy Nanoscience Institute at Berkeley, Berkeley, CA, 94720, USA*

‖*Current address: Western Regional Research Center, USDA-ARS, Albany, CA, 94710, USA*

⊥*Department of Plant and Microbial Biology, University of California, Berkeley, Berekeley, CA, 94720, USA*

#*Current address: Department of Chemistry, Columbia University, New York, 10027, USA*

@*These authors contributed equally to this work.*

E-mail: grfleming@lbl.gov

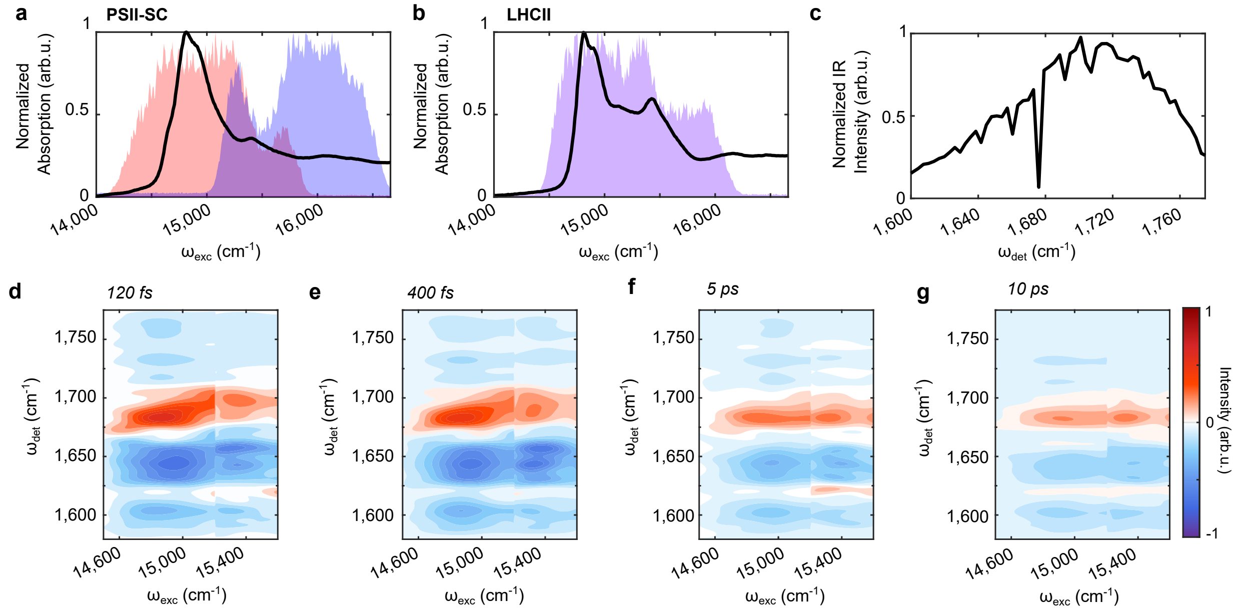

Figure S1: **Experimental spectra.** Normalized absorption spectrum and visible excitation pump spectra for the 2DEV measurements on the **(a)** PSII-SC and **(b)** isolated LHCII trimer. **(c)** Infrared probe spectrum used for all 2DEV measurements. 2DEV maps at different time delays for the PSII-SC: **(d)** 120 fs, **(e)** 400 fs, **(f)** 5 ps and **(g)** 10 ps. The excitation at 15,200 cm^{-1} marks the separation between the two PSII-SC measurements (see panel (a) and Experimental).

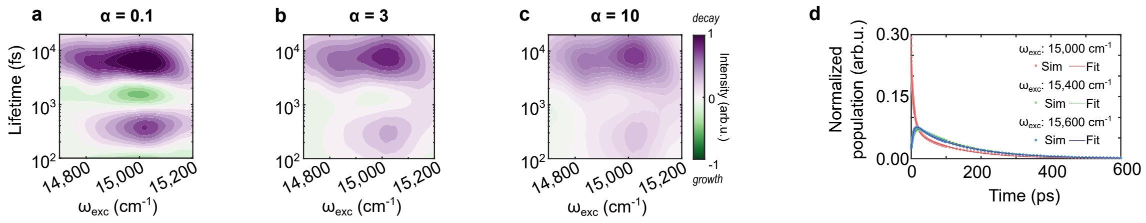

Figure S2: **Example of cross-checking of LDA analysis results.** LDM for the simulated population evolution of CP43 in the PSII-SC for hyper-parameter α of **(a)** 0.1, **(b)** 3 and **(c)** 10. With increasing α the negative amplitude around 1 ps disappears. The corresponding growth is not observed via exponential fitting of the simulated population evolution of CP43 at different excitation frequencies (see Table S3 and SI section 3). **(d)** Exponential fit of the simulated population evolution of CP43 for three different excitation frequencies (fit parameters are reported in Table S3)

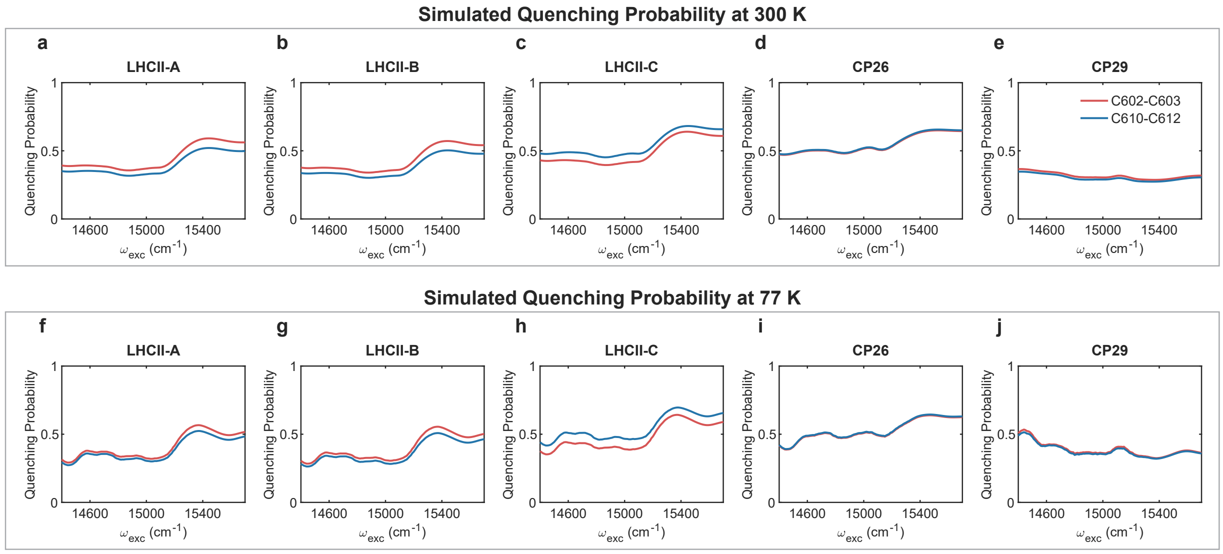

Figure S3: **Simulated quenching probability in the peripheral antennae of the PSII-SC at 300 K and 77 K.** Probability that the excitation energy is quenched before reaching the RC, with quenching sites (the Chls near carotenoids) being in **(a, f)** LHCII-A (G/g), **(b, g)** LHCII-B (N/n), **(c, h)** LHCII-C (Y/y), **(d, i)** CP26 and **(e, j)** CP29 at 300 K **(a-e)** and 77 K **(f-j)**. The EET rate from Chls to carotenoid is universally set to (200 fs)$^{-1}$ and the exact quenching sites (red: C602-C603; blue: C610-C612) are selected based on literature.[1,2] Detailed description can be found in Experimental.

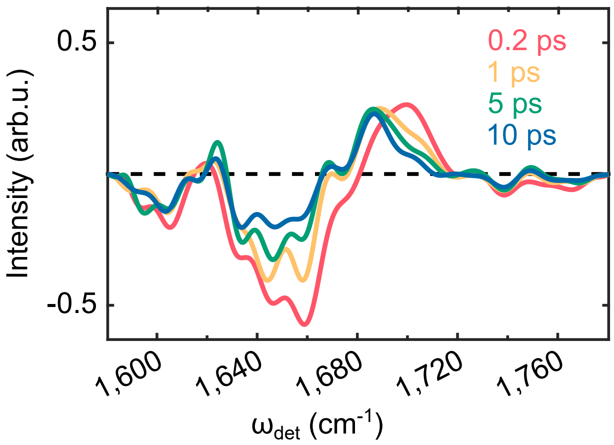

Figure S4: **An example of IR structure evolution at 15400 cm^{-1} excitation.** The evolution does not show a global decay, but rather fine structural changes. This shows that annihilation is not a complicating factor in our experiment as we would expect to see a universal decay for annihilation dynamics.[3]

1 Improved Spectral Resolution of 2DEV Spectroscopy

A detailed discussion on the origin of the improved spectral resolution of 2DEV spectroscopy can be found in ref.[4] Briefly, in pigment-protein complexes, the pigment electronic degrees of freedom are influenced by the electronic couplings with other pigments, while the pigment nuclear degrees of freedom, particularly the highly localized modes, are influenced by the interactions with the local protein residues. This indicates that, in 2DEV spectroscopy, the factors shifting the spectral responses of a pigment on the excitation axis and on the detection axis have little to no correlation. Therefore, the chances of observing a peak at a specific position of a 2DEV spectrum depends on the conditional probability $P(\omega_{exc}, \omega_{det})$, where ω_{exc} is the excitation frequency and ω_{det} is the detection frequency. In other words, the chances of having overlapping spectral responses from multiple pigments are lower compared to 2D electronic spectroscopy, where the two axes are correlated, and visible pump-IR probe spectroscopy, which does not have resolution of excitation frequency.

The improved spectral resolution of 2DEV spectroscopy has been demonstrated by the studies of smaller subunits of PSII,[5–7] where different IR structures can be clearly seen at different excitation frequencies. Figure 2 also shows some demonstrations, including the 2DEV spectral slices at different of excitation frequencies for the PSII-SC. For example, the ESA region between 1630 cm^{-1} and 1660 cm^{-1} for the PSII-SC show clear excitation frequency dependence. The ESA band at 14,700 cm^{-1} and 15,000 cm^{-1} (Figure 2a) have similar structure to the same ESA band of the PSII-CC at the two excitation frequencies (Figure 2c). On the other hand,the ESA band at 15,300 cm^{-1} and 15,600 cm^{-1} (Figure 2a) share more resemblance with the same ESA band of LHCII at the two excitation frequencies (Figure 2b), particularly the peak at 1,657 cm^{-1}.

2 Spectral Density Definitions

The spectral density parameters are listed in Table S1 and S2. Two different spectral densities were applied for the simulation to ensure consistency with the literature Hamiltonian of each protein subunit, as described by Bennett et al.[8]

For the PSII-CC components, the spectral density is defined[8,9] as

$$\chi''(\omega) = (\pi\hbar)\frac{S_0}{s_1 + s_2}\sum_{i=1,2}\frac{s_i\omega^5}{7!2\omega_i^4}e^{-\sqrt{\frac{\omega}{\omega_i}}} \tag{1}$$

where S_0, s_1, s_2, ω_1, and ω_2 are the parameters listed in Table S2.

For the minor antennae and LHCII, the spectral density is defined[8,10] as

$$\chi''(\omega) = 2\lambda_0\frac{\omega\Gamma_0}{\omega^2 + \Gamma_0^2} \tag{2}$$

where λ_0 and Γ_0 are the parameters listed in Table S2. Additionally, vibronic coupling with individual modes are also included for the peripheral antennae, which contributes to the spectral density as[8,10]

$$\chi''_{vib}(\omega) = \sum_{j=1}^{N_{vib}} 2S_j\omega_j^3\frac{\omega\Gamma_{vib}}{(\omega_j^2 - \omega^2)^2 + \omega^2\Gamma_{vib}^2} \tag{3}$$

where S_j, ω_j, and Γ_{vib} are the parameters for each vibration modes. The values of these parameters can be found in ref[8] and ref.[11]

Table S1: EET simulation parameters.

	RC[a]		CP43[b]/CP47[c]	CP26/CP29[d]		LHCII[e]	
Pigment	Chl *a*	Pheo	Chl *a*	Chl *a*	Chl *b*	Chl *a*	Chl *b*
TDM[f]	4.4	3.5	4.4	3.74	3.18	4	3.4
σ_{inhom}[g]	200	120	180	90	108	80	96
SD type[h]	a	b	c	d	e	f	g

[a] Raszewski et al.[9,12] (adapted by Bennett et al.[8])
[b] Müh et al.[13] (adapted by Bennett et al.[8])
[c] Raszewski et al.[9] (adapted by Bennett et al.[8])
[d] Mascoli et al.[11]
[e] Novoderezhkin et al.[10] (adapted by Bennett et al.[8])
[f] Transition dipole moment magnitude [unit: Debye]
[g] Inhomogeneous broadening width [unit: cm^{-1}]
[h] Spectral density type in Table S2

Table S2: Spectral density parameters (detailed description can be found in Supporting Information Section 2).

	a	b	c	d	e	f	g
S_0	0.65	0.65	0.5				
s_1	0.8	0.8	0.8				
s_2	0.5	0.5	0.5				
ω_1 [cm^{-1}]	0.532	0.532	0.532				
ω_2 [cm^{-1}]	1.94	1.94	1.94				
λ_0 [cm^{-1}]				40	48	37	48
γ_0 [cm^{-1}]				40	40	30	30
UD BO[a]	No	No	No	Yes	Yes	Yes	Yes

[a]This row indicates whether under-damped Brownian oscillators were included in the spectral density.

3 Fitting of Simulated Excitation Population Evolution of the PSII-SC Subunits

The simulated excitation population evolution of PSII-SC subunits (see Experimental) was subjected to exponential fitting with the following equation

$$f(t) = \sum_i A_i e^{\frac{t}{\tau_i}} \tag{4}$$

where $f(t)$ is the excitation population evolution (traces) of individual PSII-SC subunits, and A_i and τ_i are the variables, reported in Table S3 (Figure **S2**). The number of components lies between 2-4, decided based on the LDA results as well as the fitting quality.

Fitting is necessary for confirming the validity of LDA, as artifacts rise for too low values of the regularization parameter α (see Experimental and Figure **S2**), which influences the interpretation of both experimental and simulation results. The fittings were performed on the traces of selected excitation frequencies for each PSII-SC subunit. The excitation frequencies and initial parameters for the fittings are selected based on the LDMs obtained for $alpha = 0.1$. It is important to note that, in some cases, the traces cannot be described as the sum of a few exponential components. For example, the fitting results for CP47 traces display visible difference from the actual population evolution. Additionally, the fitting of the RC trace at 15,400 cm^{-1} shows a decaying component around 10 ps. However, the population evolution of the RC should be monotonically increasing as it is assumed that charge separation occurs as soon as energy reaches the RC (instant trapping). These issues with fitting of noiseless simulated population evolution likely arise from the nature of non-exponential dynamics within the system. Since the system contains a large number of EET pathways, the complicated network does not always guarantee an exponential dynamics. Therefore, the lifetimes obtained from exponential fitting and LDA should be treated as a reflection of characteristic timescales, instead of actual lifetimes of individual EET pathways.

Table S3: Exponential fit of simulated population evolution of the PSII-SC subunits. A detailed description can be found in Supporting Information Section 3).

	**CP43**			**CP26**		
ω_{exc} [cm^{-1}]	15000	15400	15600	15000	15400	15600
A_1	0.07	-0.06	-0.11	-0.06	0.01	0.05
τ_1 (ps)	*0.36*	*8.02*	*7.74*	*0.32*	*1.20*	*7.57*
A_2	0.15	0.09	0.05	-0.06	-0.01	0.05
τ_2 (ps)	*8.24*	*149.20*	*15.23*	*5.86*	*4.93*	*57.24*
A_3	0.07		0.08	0.10	-0.04	0.13
τ_3 (ps)	*123.20*		*147.80*	*33.18*	*20.60*	*159.30*
A_4				0.10	0.17	
τ_4 (ps)				*163.00*	*145.90*	
R^2	0.99974	0.99984	0.99974	1.00000	0.99997	0.99999

	**CP47**			**CP29**		
ω_{exc} [cm^{-1}]	14900	15300	15600	14900	15300	15600
A_1	0.04	0.04	-0.08	-0.07	-0.04	0.08
τ_1 (ps)	*6.50*	*5.71*	*11.56*	*5.90*	*6.01*	*11.71*
A_2	0.17	-0.12	-0.09	0.14	-0.18	-0.12
τ_2 (ps)	*183.40*	*73.66*	*65.21*	*170.90*	*180.20*	*108.90*
A_3		0.21	0.20		0.26	0.23
τ_3 (ps)		*175.10*	*175.40*		*156.00*	*161.30*
R^2	0.99932	0.99996	0.99998	0.99981	0.99988	0.99997

	**LHCII**			**RC**		
ω_{exc} [cm^{-1}]	15000	15400	15500	14800	15000	15400
A_1	-0.01	-0.01	0.02	-0.06	-0.10	0.04
τ_1 (ps)	*0.51*	*1.31*	*10.23*	*10.52*	*34.50*	*10.08*
A_2	-0.04	0.06	0.24	-0.80	-0.77	-1.025
τ_2 (ps)	*4.48*	*9.15*	*102.20*	*159.60*	*169.70*	*166.00*
A_3	-0.06	0.19	0.30	1.00	1.00	1.00
τ_3 (ps)	*28.57*	*70.51*	*172.10*	*3.76E5*	*3.39E5*	*3.13E5*
A_4	0.32	0.45				
τ_4 (ps)	*160.60*	*158.10*				
R^2	1.00000	1.00000	0.99999	0.99999	0.999980	1.00000

References

(1) Ballottari, M.; Mozzo, M.; Girardon, J.; Hienerwadel, R.; Bassi, R. Chlorophyll triplet quenching and photoprotection in the higher plant monomeric antenna protein Lhcb5. *The Journal of Physical Chemistry B* **2013**, *117*, 11337–11348.

(2) Ruban, A. V.; Berera, R.; Ilioaia, C.; Van Stokkum, I. H.; Kennis, J. T.; Pascal, A. A.; Van Amerongen, H.; Robert, B.; Horton, P.; Van Grondelle, R. Identification of a mechanism of photoprotective energy dissipation in higher plants. *Nature* **2007**, *450*, 575–578.

(3) Groot, M. L.; Breton, J.; van Wilderen, L. J.; Dekker, J. P.; van Grondelle, R. Femtosecond visible/visible and visible/mid-IR pump- probe study of the photosystem II core antenna complex CP47. *The Journal of Physical Chemistry B* **2004**, *108*, 8001–8006.

(4) Arsenault, E. A.; Bhattacharyya, P.; Yoneda, Y.; Fleming, G. R. Two-dimensional electronic–vibrational spectroscopy: Exploring the interplay of electrons and nuclei in excited state molecular dynamics. *The Journal of chemical physics* **2021**, *155*, 020901.

(5) Lewis, N. H.; Gruenke, N. L.; Oliver, T. A.; Ballottari, M.; Bassi, R.; Fleming, G. R. Observation of electronic excitation transfer through light harvesting complex II using two-dimensional electronic–vibrational spectroscopy. *The journal of physical chemistry letters* **2016**, *7*, 4197–4206.

(6) Yoneda, Y.; Arsenault, E. A.; Yang, S.-J.; Orcutt, K.; Iwai, M.; Fleming, G. R. The initial charge separation step in oxygenic photosynthesis. *Nature communications* **2022**, *13*, 2275.

(7) Yang, S.-J.; Arsenault, E. A.; Orcutt, K.; Iwai, M.; Yoneda, Y.; Fleming, G. R. From antenna to reaction center: Pathways of ultrafast energy and charge transfer in photosystem II. *Proceedings of the National Academy of Sciences* **2022**, *119*, e2208033119.

(8) Bennett, D. I.; Amarnath, K.; Fleming, G. R. A structure-based model of energy transfer reveals the principles of light harvesting in photosystem II supercomplexes. *Journal of the American Chemical Society* **2013**, *135*, 9164–9173.

(9) Raszewski, G.; Renger, T. Light harvesting in photosystem II core complexes is limited by the transfer to the trap: can the core complex turn into a photoprotective mode? *Journal of the American Chemical Society* **2008**, *130*, 4431–4446.

(10) Novoderezhkin, V.; Marin, A.; van Grondelle, R. Intra-and inter-monomeric transfers in the light harvesting LHCII complex: the Redfield–Förster picture. *Physical Chemistry Chemical Physics* **2011**, *13*, 17093–17103.

(11) Mascoli, V.; Novoderezhkin, V.; Liguori, N.; Xu, P.; Croce, R. Design principles of solar light harvesting in plants: Functional architecture of the monomeric antenna CP29. *Biochimica Et Biophysica Acta (BBA)-Bioenergetics* **2020**, *1861*, 148156.

(12) Raszewski, G.; Saenger, W.; Renger, T. Theory of optical spectra of photosystem II reaction centers: location of the triplet state and the identity of the primary electron donor. *Biophysical Journal* **2005**, *88*, 986–998.

(13) Müh, F.; Madjet, M. E.-A.; Renger, T. Structure-based simulation of linear optical spectra of the CP43 core antenna of photosystem II. *Photosynthesis research* **2012**, *111*, 87–101.
